# Ultra‐Stable and Highly Luminescent Perovskite for Multi‐Color Ultraviolet Single‐Pixel Imaging

**DOI:** 10.1002/advs.202504307

**Published:** 2025-04-07

**Authors:** Bin Xu, Menglu Chen, Kenan Zhang, Jianbang Mu, Jie Cao, Hongyu Lv, Haoyu Zhang, Xingting Zhou, Feng Shi, Qun Hao

**Affiliations:** ^1^ School of Optics and Photonics Beijing Institute of Technology Beijing 100081 China; ^2^ Zhejiang Key Laboratory of 3D Micro/Nano Fabrication and Characterization Westlake Institute for Optoelectronics Zhejiang 3114 China; ^3^ Laboratory of Science and Technology on Integrated Logistics Support Changsha Hunan 410073 China; ^4^ Yangtze Delta Region Academy of Beijing Institute of Technology Jiaxing 314019 China; ^5^ Physics Department Changchun University of Science and Technology Changchun 130022 China

**Keywords:** multi‐color, perovskite, single‐pixel imaging, ultraviolet

## Abstract

Ultraviolet (UV) detection and spectral imaging technology play a crucial role in information acquisition. However, this wavelength is beyond the silicon detectors’ response range. Currently, all‐inorganic lead halide perovskite nanocrystals exhibit excellent photoluminescent properties converting UV photons to visible photons, which provide a solution for UV detection. Nevertheless, their practical application is restricted by poor stability when exposed to water, UV light, or heat. Therefore, it is essential to develop a viable preparation method for producing high‐performance thin film devices. In this study, a design is proposed involving CsPbX_3_ composites, doped with Mg^2+^ and coated with Styrene‐Ethylene‐Butylene‐Styrene (SEBS). Notably, the Mg‐CsPbX_3_@SEBS demonstrate exceptional environmental stability, linearity in UV resistance, and water stability, exhibiting a mere 18% decrease in luminous intensity after 168 h of immersion, Additionally, these composites demonstrate high‐temperature stability, enduring temperatures up to 500 K. Using compressed sensing technology and composite films, multi‐color imaging is also achieved under various UV light conditions with a resolution of 128 × 128 pixels. This work provides valuable insights into the down‐conversion properties of perovskite materials, broadening their potential applications.

## Graphical abstract

In this work, a novel strategy is presented for co‐passivation of perovskites via Mg^2+^ and SEBS, achieving high PLQY and enhanced stability in perovskite films, utilizing Fourier compressed sensing technology, integrated with single‐pixel imaging technology for UV detection and imaging.

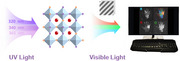

## 1 Introduction

Ultraviolet (UV) detection and spectral imaging technology play a crucial role on information acquisition.^[^ ^1^ ^]^ Currently, silicon photodetector arrays are extensively utilized in imaging, with high resolution and detectivity, including charge‐coupled devices (CCDs) and complementary metal‐oxide‐semiconductor (CMOS).^[^ ^2^ ^]^ However, due to the silicon bandgap, the spectral wavelength is limited in visible and near infrared. For non‐visible bands, such as UV and mid‐infrared, which also hold significant value, there are still challenges on cost‐effective technology for detector array fabrication.^[^ ^3^, ^4^ ^]^ Nevertheless, the single‐pixel detector technology is expected to solve this limitation. Compared to array detectors, single‐pixel detectors exhibit lower dark noise levels, enhanced quantum effects, and do not require complex optical systems within the single‐pixel imaging framework.^[^ ^5^ ^]^ The single‐pixel imaging, also called ghost imaging, involves capturing variations in the 2D light field of reflected light alongside the spatial distribution of structured light.^[^ ^6^ ^]^ By conducting autocorrelation operations, target images can be reconstructed. Single‐pixel imaging technology effectively mitigates disturbances and signal attenuation caused by scattering media, making it widely applicable in non‐visual field imaging.

In UV detection and spectral imaging technologies, the quality of the resulting images is primarily determined by the resolution of the projected patterns. Consequently, the digital micromirror devices (DMD) used for pattern projection is crucial. However, existing DMDs exhibit limited efficiency in the UV band due to less mature manufacturing processes, leading to significant signal loss and degraded imaging quality. Furthermore, the photoelectric materials directly exposed to UV light are prone to photodegradation, particularly under prolonged or high‐intensity illumination, resulting in compromised stability. Additionally, DMDs and photoelectric sensors suitable for the UV band are considerably more expensive than their visible band counterparts.^[^ ^7^, ^8^ ^]^ A promising alternative involves utilizing perovskite quantum dots (PQDs), which can dynamically adjust their absorption properties through modifications in size and chemical composition.^[^ ^9^, ^10^ ^]^ It enables the conversion of fluctuations in the 2D light field within the UV band into the visible range, thereby facilitating direct image recovery using mature visible‐range DMD and detectors. This approach not only reduces costs but also enhances image quality, representing a significant advancement in UV detection and spectral imaging.

Metal halide‐based PQDs materials exhibit exceptional optical properties, including a high absorption coefficient and an adjustable bandgap.^[^ ^11^ ^]^ These characteristics render perovskites highly efficient in converting UV light into visible light, making them ideal for achieving superior conversion efficiency. In the context of single‐pixel detectors, this efficiency translates to the capability to capture and convert optical signals effectively, even with weak luminescence, thereby presenting significant potential for applications in spectral imaging systems. However, the practical utilization of perovskites is limited by their inherent instability and susceptibility to decomposition when exposed to elevated temperatures, high humidity, and UV radiation.^[^ ^12^, ^13^ ^]^

In this study, the adjustment of the bandgap of CsPbX_3_ facilitates the realization of a complete visible light emission spectrum, spanning from blue to red. The incorporation of Mg^2+^ for surface defect passivation in CsPbX_3_ quantum dots results in an impressive photoluminescence quantum yield (PLQY) of up to 98%. Furthermore, the resultant Mg‐CsPbBr_3_@SEBS complex demonstrates a substantial PLQY of 83.6%, exhibiting superior long‐term stability against water, UV light, and heat. Mg‐CsPbX₃@SEBS not only exhibits remarkable efficiency in converting UV light into visible light but also demonstrates distinct excitation intensities under different UV spectral bands. Furthermore, its exceptional stability ensures high reliability for prolonged use in UV imaging applications. By integrating a ghost imaging system with Mg‐CsPbX_3_@SEBS and employing a compressed sensing algorithm, we achieve high‐resolution and high‐contrast multi‐color images with a resolution of 128 × 128 pixels. This technology enables UV light detection and spectral imaging, even under conditions of weak UV illumination. Additionally, this technology holds promising applications in invisible light detection, image encryption, and anti‐counterfeiting, among others.

## 2 Results and Discussion

**Scheme 1:**
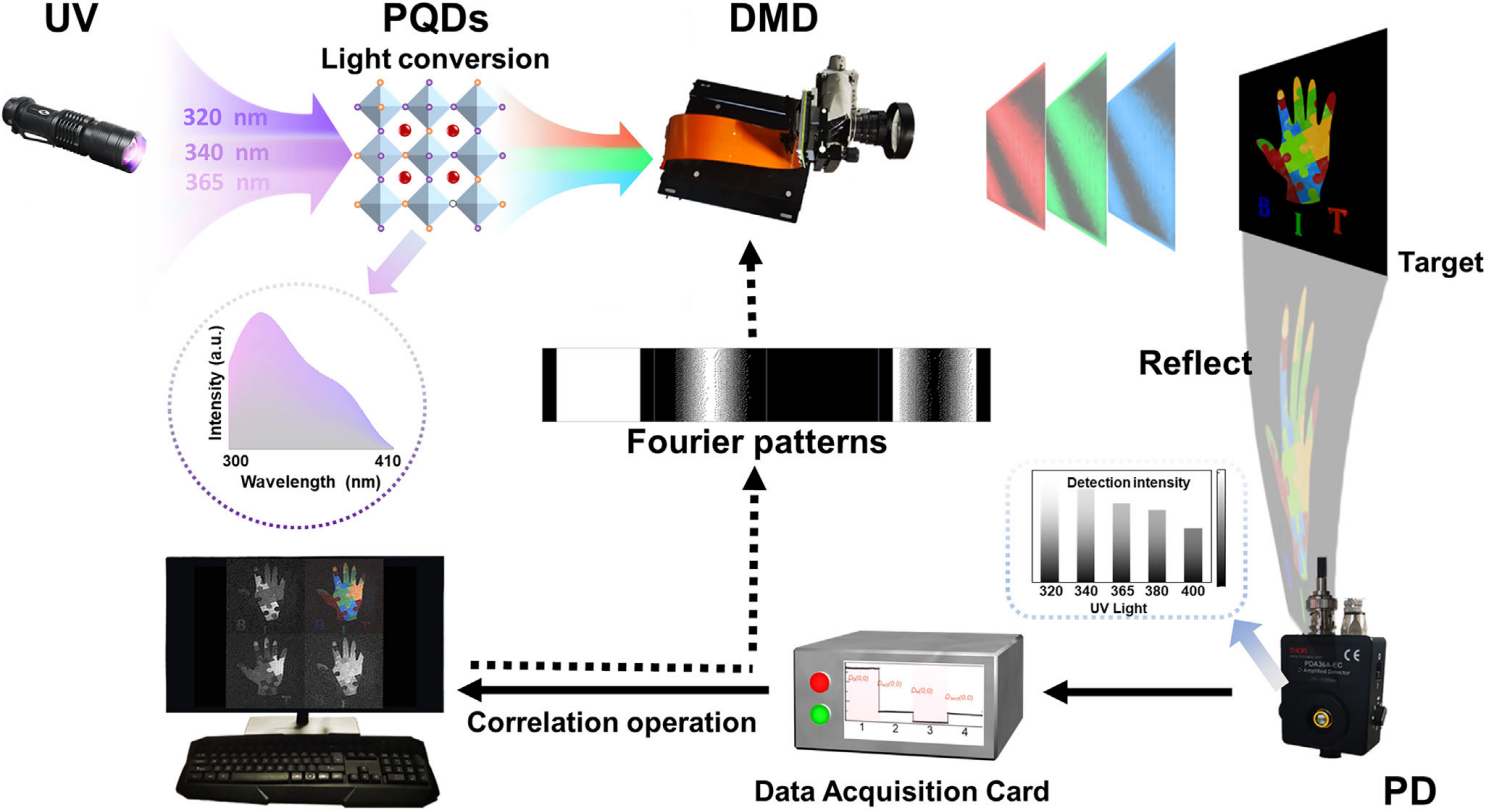
Single‐pixel imaging design based on perovskite materials.

In this study, we develop a single‐pixel multi‐color imaging system that utilizes perovskite materials. As illustrated in **Scheme** **1** and Figure S1 (Supporting Information), the illumination of the system is achieved by using different UV lamps. As shown in Figure S2 (Supporting Information). These PQDs composite materials exhibit a broad excitation range within the UV spectrum. The photoluminescence (PL) peak position of the PQDs composite materials have no excitation dependence, while the PL intensity under different UV excitation is obviously different (Figure S3, Supporting Information). The PL intensity variations are then encoded by a DMD to modulate both the spatial and spectral characteristics of the object. The overall signal is recorded by a single pixel silicon detector. By correlating the recorded light intensity with the spatial and spectral modulation patterns on the DMD, we can effectively reconstruct high‐resolution images of the object while obtaining detailed spectral information for each pixel in the image.

**Figure 1:**
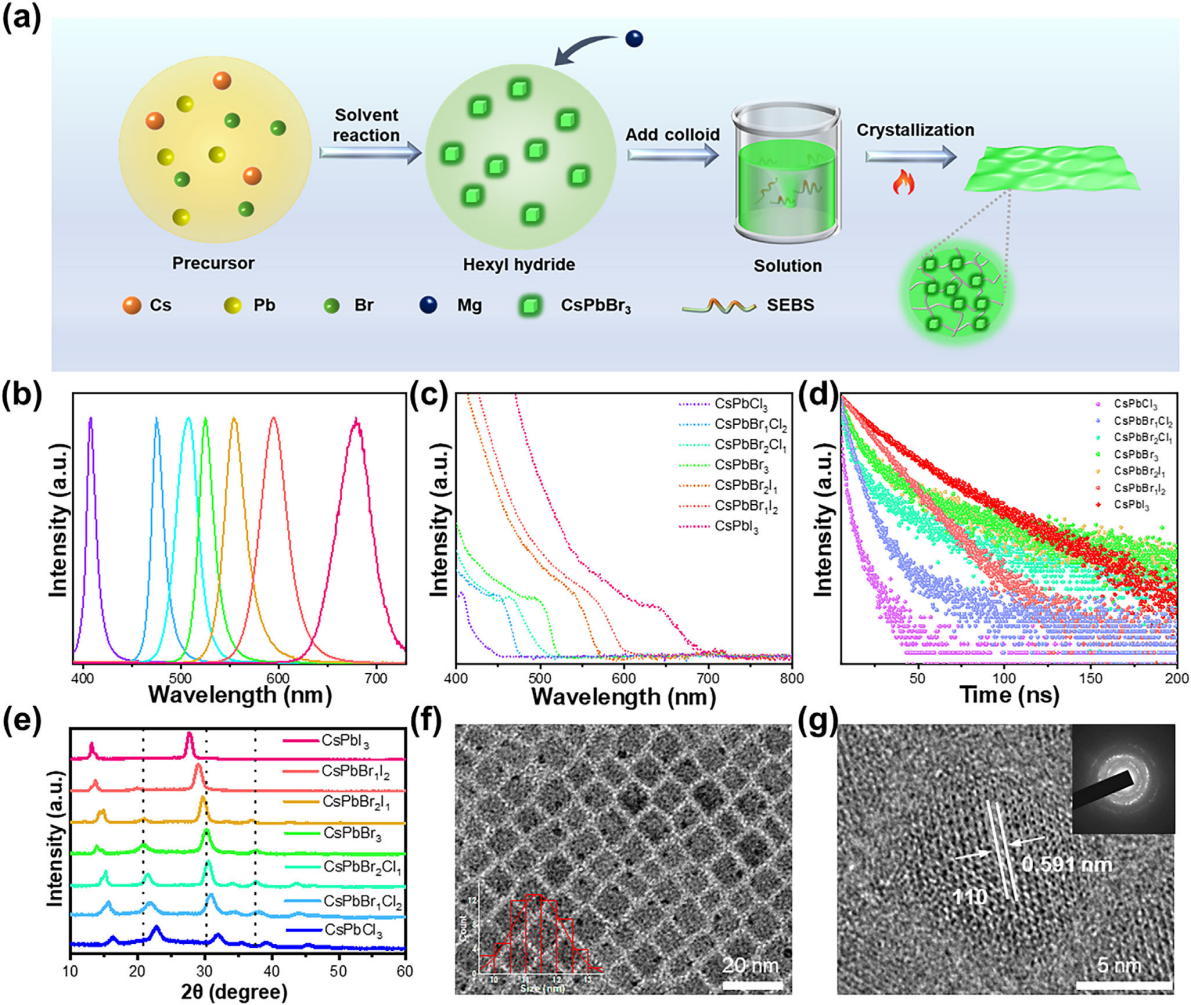
Schematic diagram of the manufacturing process and morphological characterization of CsPbBr_3_ quantum dots and Mg‐CsPbBr_3_@SEBS films. a) Synthesis flowchart for the synthesis of CsPbBr_3_ PQDs and Mg‐CsPbBr3@SEBS films. b) Tunable PQDs are composed of PL emission spectra, c) absorption spectra, and d) time‐resolved PL spectra. e) XRD of the tunable composition of PQDs. f) TEM image ofCsPbBr_3_ QDs. g) TEM image ofCsPbBr_3_ PQDs at high magnification.

As shown in **Figure** **1**a, high‐quality Mg^2+^‐doped lead halide PQDs were synthesized using the ligand‐assisted reprecipitation method, resulting in excellent stability and high luminous intensity. The CsPbX_3_ PQDs obtained exhibit remarkably bright PL under UV excitation. The PL emission spectrum of the PQDs demonstrates a panchromatic color tuning from purple to deep red across the entire visible spectral region, with a half‐peak full width ranging (FWHM) from 9 to 31 nm. The optical absorption spectra indicates that as the halide composition changes from Cl to I, the PQDs exhibit significant absorption in both the UV and visible regions, with the absorption band edge shifting from 408 to 692 nm (Figure 1b,c). The corresponding time‐resolved PL decays an effective lifetime ranging from 7.3 to 112.3 ns, with faster attenuation observed for wide‐bandgaps PQDs (Figure 1d; and Table S1, Supporting Information). Structural and morphological characterization, conducted through transmission electron microscopy (TEM) analysis, powder X‐ray diffraction (XRD) measurements, energy‐dispersive X‐ray spectroscopy analysis, and selected electron diffraction observations, confirm the cubic phase nature and high crystallinity of PQDs particles, which have an average length ranging from 10.8 to 12.4 nm (Figure 1e,f; Figure S4, Supporting Information).

**Figure 2:**
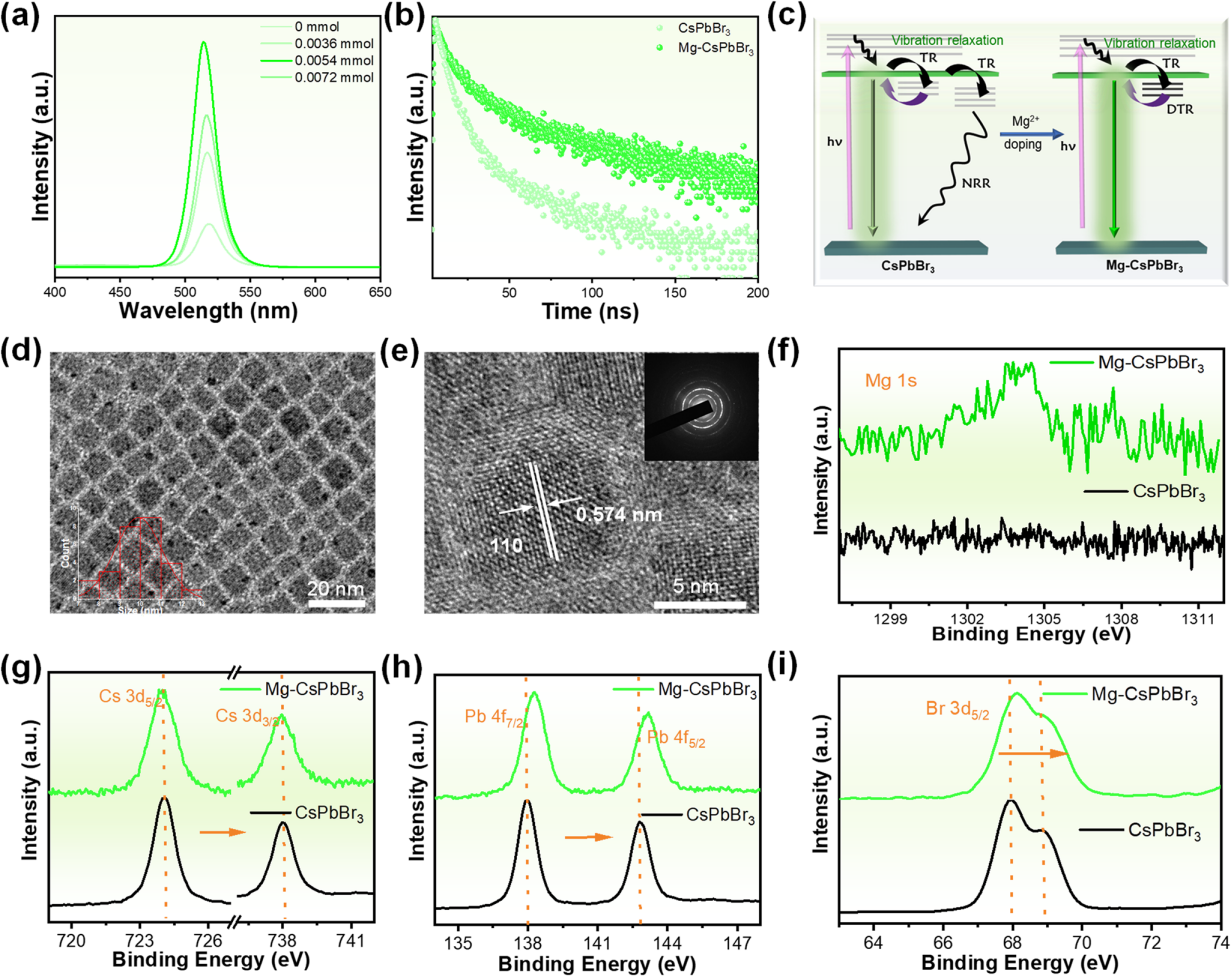
Performance Characterization of Mg‐CsPbBr_3_ PQDs. Effect of doping on a) the PL spectra and b) the time‐resolved PL spectra. c) Schematic Illustration of Different Photophysical Processes in Undoped and Doped CsPbBr_3_ NCs. d) TEM image of Mg‐CsPbBr_3_ PQDs. e) TEM image of Mg‐CsPbBr_3_ PQDs at high magnification. High‐resolution XPS spectra of the undoped and doped CsPbBr_3_ PQDs showing signals due to f) Mg 1s, g) Cs 3d, h) Pb 4f, and i) Br 3d.

The surface defects of CsPbX_3_ PQDs are passivated by Mg^2+^ ions, thus improving the emission efficiency. Notably, with increasing Mg^2+^ ion concentration in the CsPbBr_3_, both emission and absorption peaks exhibit a blue shift, likely attributed to lattice shrinkage induced by the smaller Mg^2+^ ions. The red CsPbI_3_ and blue CsPbBr_1_Cl_2_ also exemplify this phenomenon (**Figure** **2**a; Figures S5 and S6, Supporting Information). Furthermore, the PL intensity of the system is significantly enhanced.^[^ ^14^, ^15^, ^16^ ^]^ As shown in Table S2 (Supporting Information), the PLQY of CsPbBr_3_ PQDs increases from ≈64% to nearly 100%. For CsPbI_3_, it rises from ≈69% to ≈96%. Additionally, the PLQY of CsPbBr_1_Cl_2_ PQDs experiences an increase from roughly 50% to ≈87%.

**Figure 3:**
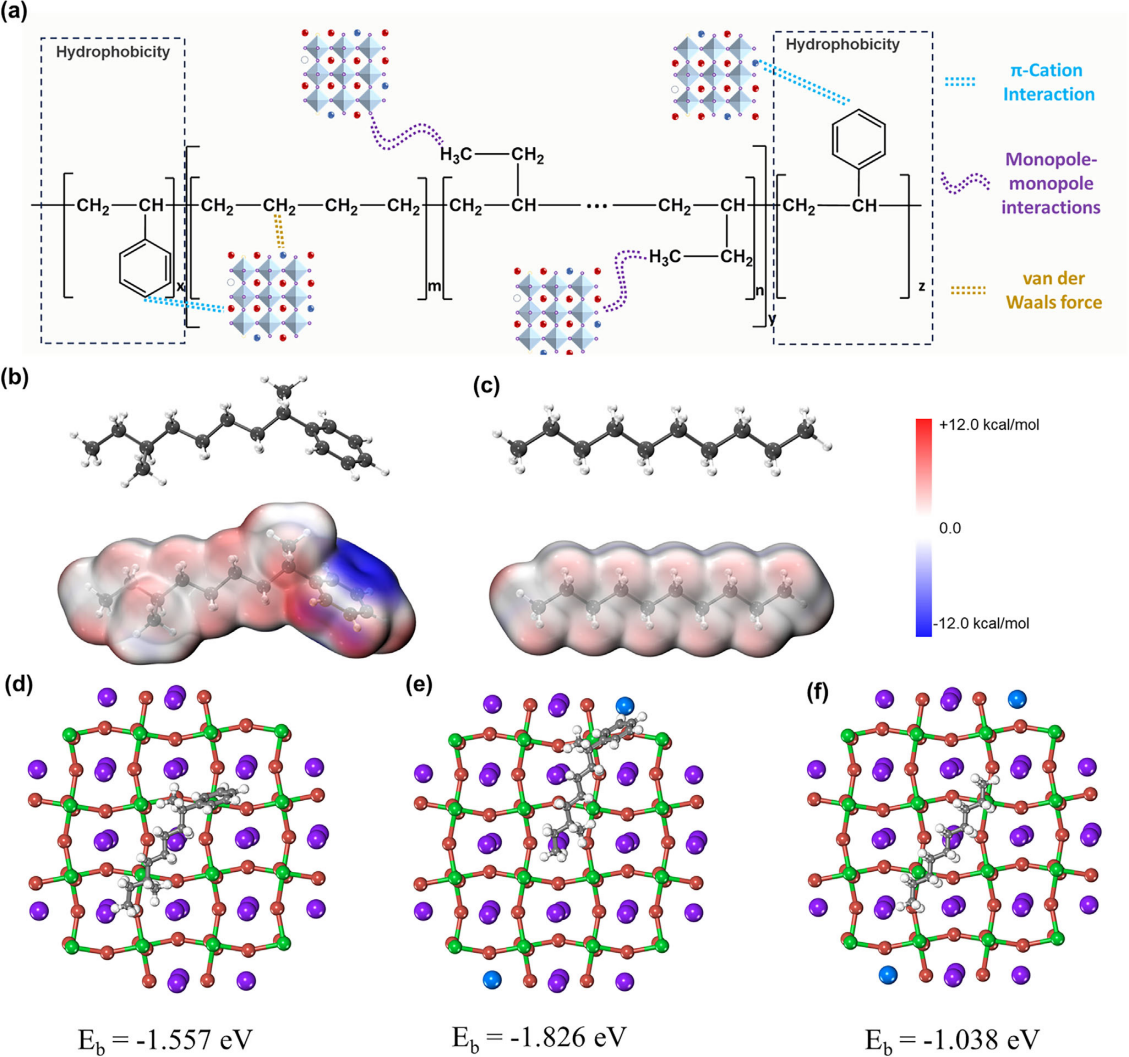
Performance Characterization of Mg‐CsPbBr3@SEBS Film. a) Abundant interactions in the Mg‐CsPbBr_3_@SEBS Film films. b) Calculated electrostatic potential profile of Styrene‐Ethylene‐Butylene‐Styrene. c) Calculated electrostatic potential profile of polyethylene. d) Differential charge density distribution of d) SEBS adsorbed on the CsPbBr_3_ surface, e) SEBS adsorbed on the Mg‐CsPbBr_3_ surface f) PE adsorbed on the Mg‐CsPbBr_3_ surface and corresponding adsorption energy.

The time‐resolved PL decay curves of both undoped and Mg^2+^‐doped CsPbBr_3_ PQDs are illustrated in Figure 2b. The PL decay curves were fitted by a biexponential function: *I*(t) = *A*_1_exp($\frac{-t}{\tau_{1}}$) +*A*_2_exp($\frac{-t}{\tau_{2}}$), Generally, the short τ_1_ and longer τ_2_ components are associated with the recombination of free and bound excitons trapped on the surface. The average PL lifetime is calculated as τ_ave_ =$\;\frac{A_{1}\times \tau_{1}^{2}+A_{2}\times \tau_{2}^{2}}{A_{1}\times \tau_{1}+A_{2}\times \tau_{2}}$, The fitting and calculated results are displayed in Figure S7 (Supporting Information) and Table S3 (Supporting Information). The addition of Mg^2+^ results in an enhancement of the short lifetime component's weight (A_1_) and a corresponding decrease in the weight of the long lifetime component (A_2_), indicating a reduction in surface defect states. When the PQDs are excited by appropriate photons, electrons are stimulated to transition from the valence band (VB) to the conduction band (CB). This process is followed by the recombination of electrons and holes, resulting in light emission. However, non‐radiative pathways would diminish the PLQY of the PQDs (Figure 2c). Mg^2+^ dopants can reduce carrier trapping in both in‐band or inter‐band defect states, thereby enhancing radiative carrier recombination and promoting in‐band carrier coupling.^[^ ^17^ ^]^ Consequently, it results in improved luminescence properties of Mg^2+^ in doped CsPbX_3_ PQDs. The morphology of Mg‐CsPbX_3_ was examined using TEM. TEM images and size distribution analyses of Mg‐CsPbX_3_ PQDs are presented in Figure 2d,e, respectively. Energy‐dispersive spectrometry (EDS) elemental mappings presented in Figure S8 (Supporting Information) shown the successful incorporation of Mg^2+^ ions into CsPbBr_3_ PQDs. The surface spacing of undoped CsPbBr_3_ is measured to be 0.591 nm, which is consistent with the results reported in the literature.^[^ ^18^ ^]^ In contrast, Mg‐CsPbBr_3_ exhibits a significantly reduced interfacial distance of 0.574 nm, primarily attributed to the smaller ionic radius of Mg^2+^ compared to Pb^2+^, leading to lattice contraction. as shown in Figure S9 (Supporting Information), the shift of the diffraction peaks toward higher angles further corroborates this observation. With the increasing content of Mg^2+^, the slight variation in nanoparticle size leads to spectral shifts and changes in optical properties, which can be attributed to the relatively weak quantum confinement effect in Mg^2+^‐doped nanoparticles. This is further demonstrated by the examples of Mg‐CsPbBr_1_Cl_2_ and Mg‐CsPbI_3_, as illustrated in Figure S10 (Supporting Information). Consequently, even minor variations in the size of PQDs can lead to spectral shifts and alterations in optical properties. The high‐resolution X‐ray photoelectron spectroscopy (XPS) spectra displayed in **Figure** **3**f–i represents the Mg 2p, Cs 3d, Pb 4f, Br 3d, and of both CsPbBr_3_ and Mg‐CsPbBr_3_ PQDs. Calibration with C 1s was conducted for the chemical states of Cs^+^, Pb^2+^, I^−^, and Mg^2+^ present in the PQDs. The findings indicated a distinct XPS signal for Mg^2+^ in the Mg‐CsPbBr_3_ PQDs, whereas such a signal was absent in the CsPbBr_3_ PQDs. The peak observed at 1304.6 eV in Mg‐CsPbBr_3_ PQDs can be attributed to Mg 2p, providing further confirmation that Mg^2+^ have been successfully doped into the PQDs. (Figure 2f). It was noted that the Cs^2+^ peak in Mg‐CsPbBr_3_ PQDs remain unshifted compared to that in CsPbBr_3_ PQDs (Figure 2g). The prominent Pb 4f 7/2 and Pb 4f 5/2 peaks, located at ≈138.1 and 143 eV, respectively (Figure 2h), experience a binding energy shift toward higher values by ≈0.2 eV. Additionally, the Br 3d 5/2 peak (Figure 2i) undergoes a blue shift of ≈0.2 to ≈68.4 eV in doped CsPbBr_3_ PQDs, indicating alterations in the coordination environment of Pb^2+^ and Br^−^.

To enhance the stability of PQDs, they were integrated into a superhydrophobic polymer matrix composed of polystyrene‐ethylene‐butene‐styrene (SEBS). By adjusting the halogen content, we successfully synthesized a flexible film with PL emission tunable from 461 to 671 nm. Notably, the film exhibited a PLQY exceeding 80% for the three primary colors—red, green, and blue (Figure S11, Supporting Information). Additionally, the films demonstrated narrowband emission, with full width at half maxima (FWHM) of 18, 17, and 46.5 nm for the respective colors (Figure S12, Supporting Information). To evaluate environmental stability, both PQDs and the films were exposed to ambient conditions at 25 °C with a relative humidity of 60–70%, and the absolute PL intensity was measured over time (Figure S13, Supporting Information). Due to the detrimental effects of oxygen and moisture, the PL intensity of CsPbBr_3_ gradually decreased by 24% after 60 days. In contrast, the CsPbBr_3_@SEBS film, with a thickness of only 3 µm (Figure S14, Supporting Information), showed a mere 8% reduction in PL intensity, indicating significantly enhanced stability. Given the critical importance of UV stability in devices such as LEDs and displays, UV stability testing was conducted using a 365 nm LED lamp with an intensity of 15 mW cm^−^^2^ (Figure S15, Supporting Information). The PL stability of the CsPbBr_3_@SEBS film was markedly improved, retaining up to 89% of its initial PL intensity. These results demonstrate the exceptional long‐term stability of the Mg‐CsPbX_3_@SEBS composite films, which can be attributed to the protective effects of the SEBS matrix on the Mg‐CsPbX_3_ PQDs.

To further investigate the luminescent properties and mechanisms of the films, carrier dynamics were analyzed using temperature‐dependent PL spectroscopy (Figure S16, Supporting Information). Over a temperature range of 180 to 400 K, the Mg‐CsPbBr_3_@SEBS films consistently exhibited enhanced PL intensity and reduced FWHM, indicating that the encapsulation significantly improved the thermal stability of the PQDs. To further explore the thermal stability of Mg‐CsPbBr_3_@SEBS, temperature‐dependent PL spectra were recorded in situ during multiple heating and cooling cycles between 298 and 380 K (Figure S17, Supporting Information). The observed decrease in PL intensity at elevated temperatures can be attributed to thermal activation trapping, a phenomenon that is theoretically reversible upon cooling.^[^ ^19^ ^]^ Remarkably, Mg‐CsPbBr_3_ exhibited a significant reduction in PL emission intensity after the heating and cooling cycles. In contrast, Mg‐CsPbBr_3_@SEBS showed minimal PL intensity loss at higher temperatures and nearly fully recovered upon cooling to room temperature. This observation suggests that the dual encapsulation barrier provides substantial protection against the formation of new defects in CsPbBr_3_ PQDs, effectively restricting exciton dissociation. The synergistic effects of Mg^2+^ passivation and SEBS isolation enhance the luminous recombination rate, brightness, and PLQY. Notably, the SEBS‐encapsulated Mg‐CsPbX_3_ PQDs also exhibited remarkable luminescence stability in aqueous environments. While the fluorescence of Mg‐CsPbBr_3_ PQDs was completely quenched within 5 min in water, the Mg‐CsPbBr_3_@SEBS film maintained excellent luminescent properties even after immersion in water for up to 250 h (Figure S18, Supporting Information). Analysis of PL intensity revealed only an 18% reduction in luminescence strength, highlighting the outstanding water stability of this membrane material. Moreover, the Mg‐CsPbBr_3_@SEBS films demonstrated exceptional photoluminescence stability under harsh conditions of strong acids and alkalis, as verified by adjusting the pH of the aqueous solution (Figure S19, Supporting Information). This advancement significantly broadens the potential applications of luminescent films.

In this study, Mg‐CsPbBr_3_ was synthesized and incorporated into a SEBS matrix to form a composite material based on abundant intermolecular interactions, thereby endowing it with remarkable flexibility and photoluminescence properties (Figure 3a). These interactions include Cation‐π interactions between Pb^2+^ and Mg^2+^ in Mg‐CsPbBr_3_ and the styrene units in the SEBS chains,^[^ ^20^ ^]^ monopole‐monopole interactions between the ─CH₃ groups in the SEBS chains and the Br atoms in Mg‐CsPbBr_3_,^[^ ^21^, ^22^ ^]^ as well as the rich van der Waals forces between the SEBS chains and the ‐CH_2_‐ groups. These extensive molecular interactions promote significant adhesion at the interface between Mg‐CsPbBr_3_ and the SEBS matrix. Figure 3b,c shows the optimized geometric configurations of the polymer monomers and their respective electrostatic potentials. As shown in Figure 3b, the electron‐rich aromatic rings of the styrene units in SEBS exhibit negatively charged electron clouds, while Pb^2+^and Mg^2+^ exposed on the surface of CsPbBr_3_ carry a positive charge. This inherent charge asymmetry facilitates electrostatic interactions between the π‐electron clouds of the benzene rings and the Pb^2+^ and Mg^2+^. Additionally, *π*–*π* stacking interactions between the benzene rings within SEBS molecules further enhance intermolecular stability. These interactions not only improve the stability of the composite material but also influence its optical properties. Figure 3c demonstrates that polyethylene (PE) chains, entirely composed of non‐polar C─H bonds, result in a uniform electron density distribution without localized negative charges. In contrast to materials with polar functional groups or π‐conjugated systems (such as SEBS), PE lacks electron‐rich regions or negatively charged sites to promote electrostatic interactions with Pb^2+^ and in Mg^2+^ CsPbBr_3_.

To investigate the influence of different polymers on perovskite, density functional theory (DFT) was employed to analyze the interactions between the polymers and the perovskite. The calculated charge density differences (Figure 3d,e) reveal that SEBS exhibits stronger binding strength with Mg‐CsPbBr_3_ (E_ads_ = −1.83 eV) compared to CsPbBr_3_@SEBS (E_ads_ = −1.56 eV). This enhanced interaction can be attributed to the doping of Mg^2+^, which provides additional structural sites to passivate the inherent Pb vacancies in the perovskite, whereas PE lacks electron‐rich regions (Figure 3f), thereby reducing its adsorption energy. It is important to note that the above calculations are based on interactions between perovskite and individual molecules and do not account for interactions between organic molecules. Fourier‐transform infrared spectroscopy (FTIR) was used to analyze the stretching and in‐plane bending vibrations of the C─H bonds in the benzene rings of SEBS, with peaks observed at 698 cm⁻¹. In Mg‐CsPbBr_3_@SEBS, the strong electron‐withdrawing effect of Pb^2+^/Mg^2+^ causes a redshift in the C─H bond vibration peak, providing evidence of the binding between SEBS and PQDs rather than mere surface adsorption of SEBS on the PQDs (Figure S20, Supporting Information).

**Figure 4:**
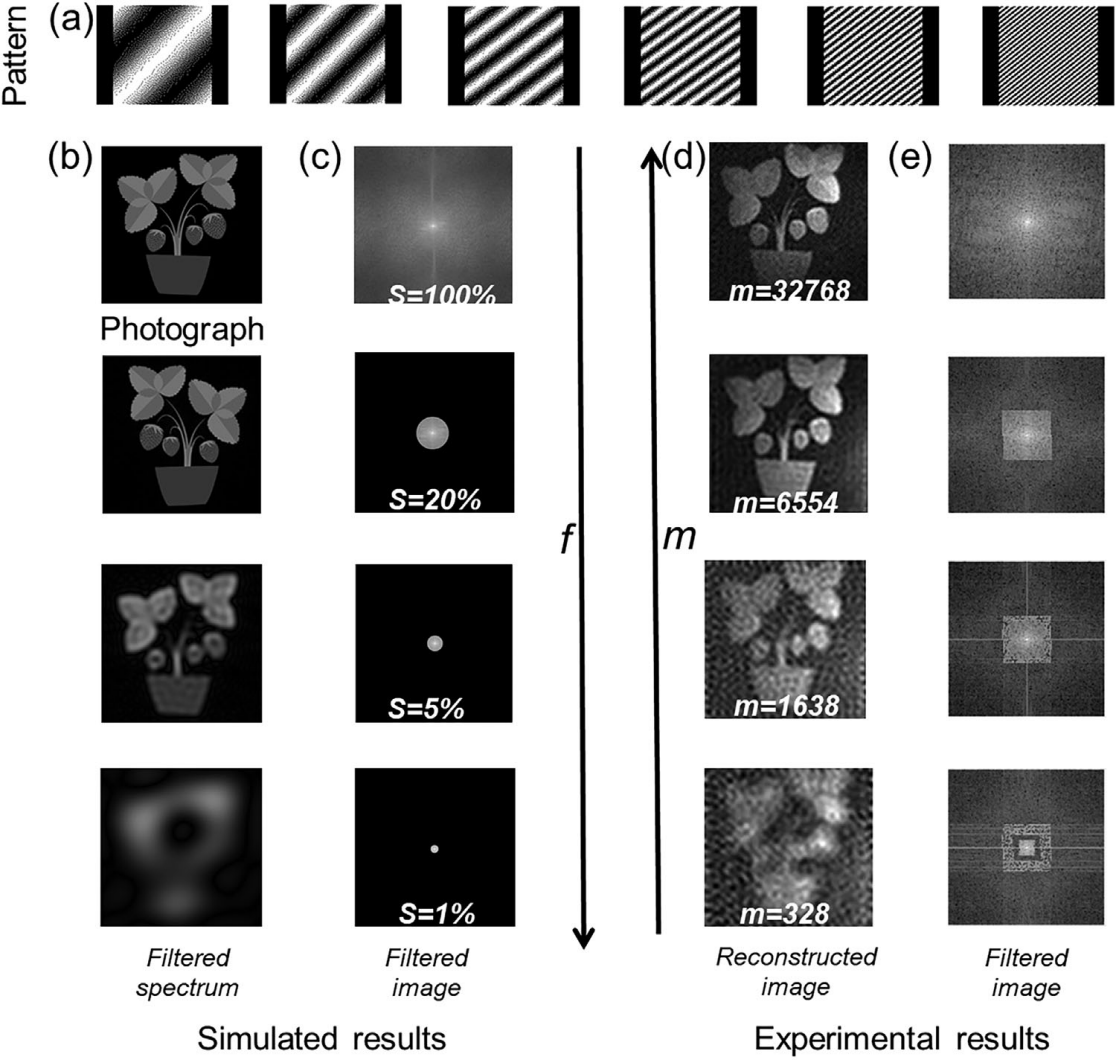
High‐resolution imaging results. a) Typical illumination patterns with a 128 × 128 resolution as spectrum coverage increases (m). b,c) The analog images correspond to varying proportions of spectrum acquisition and its spectrum. d,e) The results of experiments conducted with varying speckle numbers and their corresponding spectral graphs have been obtained.

We employed the Mg‐CsPbX_3_@SEBS film to reconstruct the target irradiated by 365 nm UV light. The following combination formula: *P*_ϕ_(*x*,*y*; *f_x_*,*f_y_*)  =  *a* + *b* · *cos*(2π*f_x_x* + 2π*f_y_y* + φ) was utilized to generate the Fourier speckle necessary for the experiment (**Figure** **4**a).^[^ ^23^ ^]^ This process requires four measurements, as each Fourier coefficient necessitates a four‐step phase‐shifted Fourier fundamental graph projection (Figure S21, Supporting Information). Given that the mathematical representation of an image is a real‐valued matrix, and its Fourier spectrum exhibits conjugate symmetry (Figure S22, Supporting Information), Images at full sampling ratio require m = 2 × I × J measurements, where I and J refer to the resolution. As shown in Figure 4b, we progressively increased the number of speckles from 328(1% × 2 × 128 × 128) to 32768 (2 × 128 × 128), designating the reflected light intensity of each speckle irradiated onto the target as *D*_ϕ_(*f_x_*,*f_y_*). Coupled with the formula: *F* {*R*(*x*, *y*)} =  [*D*_0_(*f_x_*,*f_y_*) − *D*_π_(*f_x_*,*f_y_*)] + *j* · [*D*_π/2_(*f_x_*,*f_y_*) − *D*_3π/2_(*f_x_*,*f_y_*)], the collected *D*_ϕ_ increases in tandem with the acquisition rate of frequency domain information from 1% to 100% (Figure 4c), achieving full sampling and successfully reconstructing target images. Based on the speckle information, it is evident that as the collection rate increases, the sparse streaks within the speckles become tighter, and higher‐frequency details are captured more effectively, leading to a gradual improvement in the quality of the restored images, as depicted in Figure 4d.^[^ ^24^ ^]^ Although noise exists at certain frequencies within the experimental environment, it is discernible from Figure 4e that the corresponding frequency domain related to the subject matter gradually diminishes, aligning well with the experimental results. The final restored image full sampling number (m = 32 768) is indeed the clearest.^[^ ^25^ ^]^ However, this method necessitates an excessively large number of speckles. The processing and calculation of the reflected light field intensity associated with such a high number of speckles will considerably extend time, even for high‐performance servers. As a result, this significantly undermines the timeliness of single‐pixel imaging, which contradicts our intended objectives. We propose the utilization of the compressed sensing algorithm. During the process of speckle generation, a phase shift occurs between speckles, represented as φ  =  2*k*π/*N*. Subsequently, we provide three formulas for computing Fourier spectral coefficients at N = 2, N = 3, and N = 4, respectively, where$\;{\tilde{I}}_{N}\left(f_{x},f_{y}\right)$ denotes the n‐step phase‐shift Fourier spectrum of the recorded image.^[^ ^26^, ^27^, ^28^ ^]^

(1) $${\tilde{I}}_{2}=\left[D_{0}-D_{\pi /2}\left(0,0\right)\right]+j\cdot \left[D_{\pi /2}-D_{\pi /2}\left(0,0\right)\right]$$

(2) $${\tilde{I}}_{3}=\left[2D_{0}-D_{2\pi /3}-D_{4\pi /3}\right]+j\cdot \left[D_{\frac{2\pi }{3}}-D_{\frac{4\pi }{3}}\right]$$

(3) $${\tilde{I}}_{4}=\left[D_{0}-D_{\pi }\right]+j\cdot \left[D_{\pi /2}-D_{3\pi /2}\right]$$

The output value of the single pixel detector at the coordinate (0, 0) when *D*_π/2_ is applied can be represented as P_π/2_(*x*,*y*,  *f_x_* =  0,  *f_y_* =  0). In practical non‐ideal experiments, the equation *D*_ϕ_(*f_x_*,*f_y_*)  = *D_n_*  + *kE*_ϕ_(*f_x_*,*f_y_*), as illustrated in Equations (1)–(3), demonstrates that the four‐step phase shift method with N = 4 effectively mitigates noise from ambient illumination due to its subtraction symmetry, thereby enhancing the signal‐to‐noise ratio of the image.^[^ ^29^ ^]^ The target reconstruction spectrum, utilizing a 20% sampling ratio, effectively masks background noise in the environment, thereby facilitating the acquisition of an image with reduced noise through the algorithm's noise suppression feature. By employing this 20% sampling ratio, we maximize data utilization, decrease both the duration of sampling and the time required for sampling, while ensuring that the restoration results remain clear.

**Figure 5:**
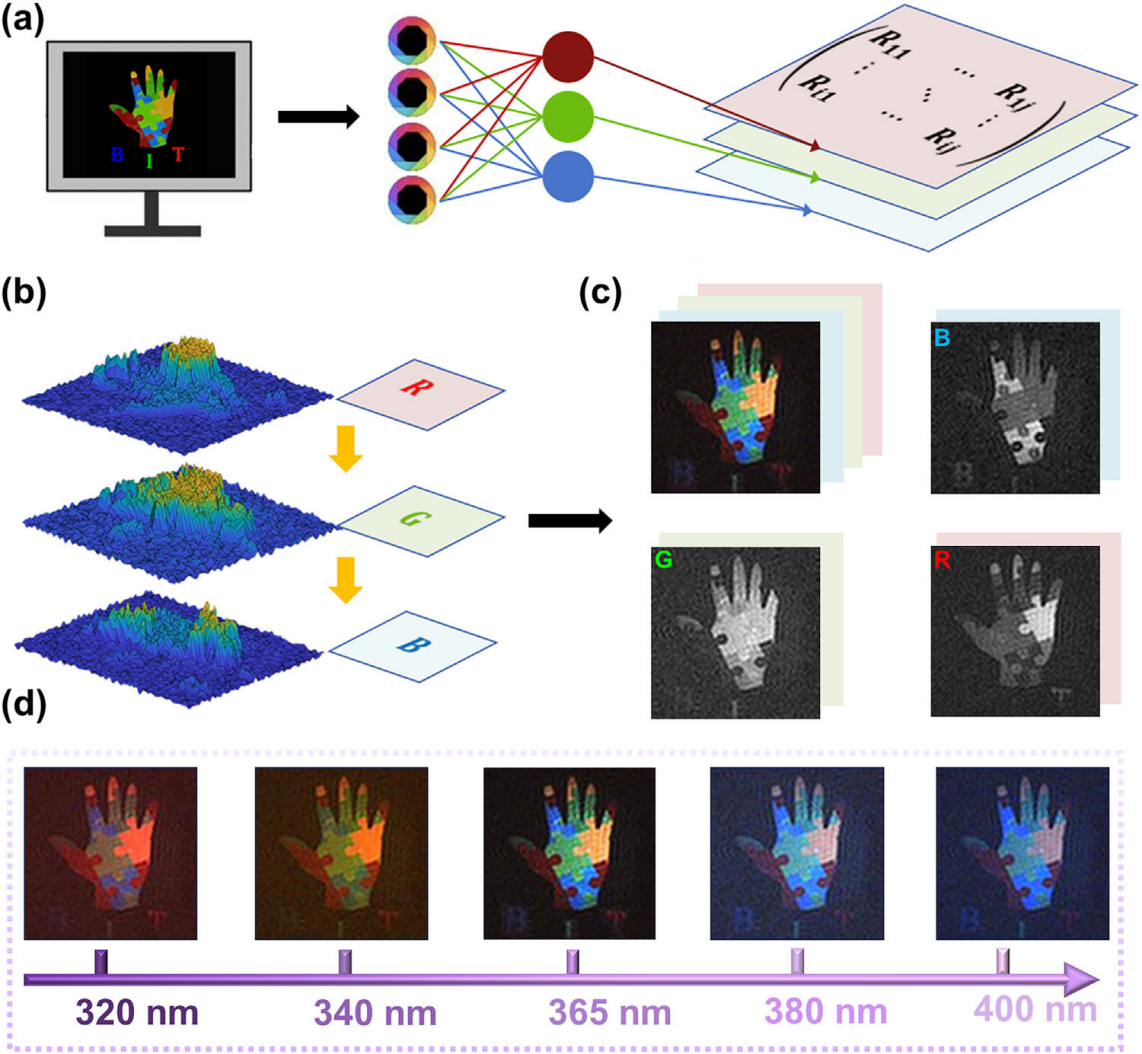
Color multispectral imaging results. a) Image's primary color is extracted and then the compressed sensing algorithm is employed to reconstruct the image. b) Intensity distribution of different layers under 365 nm irradiation. c) The layers are combined to generate the ultimate encrypted image. d) Multi‐color UV single‐pixel imaging results under different UV excitation light.

After utilizing the Mg‐CsPbX_3_@SEBS film, which exhibits efficient down‐conversion characteristics, UV light undergoes conversion into trichromic light for the reconstruction of a palm target consisting of diverse color blocks, as depicted in **Figure** **5**a. The resultant intensity distribution of trichromic layers within the image is obtained and illustrated in Figure 5b. Different images encompass entirely distinct color information.^[^ ^30^ ^]^ Upon converting the intensity into an image, various colors become visible when illuminated, thereby enabling the extraction of additional picture information corresponding to colors from the color target (Figure 5c). The imaging system has a resolution of 128 × 128 pixels and an SNR of 26.78 dB. As shown in Figures S2 and S3 (Supporting Information), Mg‐CsPbX_3_@SEBS film show unique nonlinear dependence on UV wavelength and linear dependence on UV intensity at certain UV wavelength since the PLQY is a constant. The optimal excitation wavelengths for the PQDs composite film are 385 nm at 462 nm, 370 nm at 519 nm, and 320 nm at 676 nm. One could see these CsPbX_3_@SEBS films enable the achievement of varying intensities under different UV excitation conditions, allowing multi‐color UV imaging. Figure 5d showed the successful implementation of multi‐color single‐pixel imaging using various UV light sources from 320 to 400 nm, where the visible color and intensity both vary with UV wavelength. Notably, under the 365 nm UV light, the imaging results for blue‐green and red are relatively well balanced. By analyzing the single‐pixel results alongside the characteristics of the composite materials, it is evident that different UV light detection and spectral imaging outcomes can be attained. Moreover, leveraging the color differences of the trichromic light, we demonstrate spatially resolved anti‐counterfeiting capabilities. As illustrated in Figure 5c, below the palm target, blue, green, and red letters “B,” “I,” and “T” are embedded. When illuminated with blue light, only the letter “B” is discernible, whereas red light reveals only the letter “T.” By encrypting the images obtained under single‐color illumination, the complete letters “BIT” can only be displayed when the correct algorithmic mechanism is applied for superposition, thereby achieving secure information encryption.

## 3 Conclusion

In summary, Mg^2+^ doping and SEBS surface coating were utilized in this work to synthesize Mg‐CsPbX_3_@SEBS composites that exhibit exceptional stability against water, UV light, and heat. The incorporation of Mg^2+^ enhances the PLQY of both CsPbBr_3_ and Mg‐CsPbX_3_@SEBS systems. The hydrophobic SEBS surface coating significantly increases the stability of PQDs. By leveraging the high PLQY characteristics of the Mg‐CsPbX_3_@SEBS film in conjunction with a single‐pixel imaging system, indirect imaging and detection of UV light can be accomplished. Moreover, a color imaging system for UV light is realized through the application of multi‐color tunable properties and compressed sensing technology. This research paves the way for advancements in UV light imaging and detection, potentially inspiring future applications in various optoelectronic devices.

## Conflict of Interest

The authors declare no conflict of interest.

## Author Contributions

B.X. and M.C. contributed equally to this work. B.X. and M.C. conceived and designed the idea. B.X., M.C., and J.C. designed the experiments. X.B., J.M., H.L, and H.Z. collected and analyzed the data. K.Z. and. H.L. assisted with the experiments and characterizations. B.X. and M.C. wrote the manuscript. M.C., B.X., J. M., J.C., H.Z., F.S., and Q.H. discussed the results and prepared the manuscript. All the authors reviewed and contributed to this paper.

## Supporting information



Supporting Information

## Data Availability

The data that support the findings of this study are available from the corresponding author upon reasonable request.
