# Supplementary material for: Ultra‐Stable and Highly Luminescent Perovskite for Multi‐Color Ultraviolet Single‐Pixel Imaging
Source: Adv Sci (Weinh). 2025 Apr 7;12(26):2504307. doi: 10.1002/advs.202504307 (PMC12245018; doi:10.1002/advs.202504307)
Supplement: Supplementary file 1 — Supporting Information [file ADVS-12-2504307-s001.docx]

·Supporting Information

Ultra-Stable and Highly Luminescent Perovskite Films Application for UV single-pixel imaging

Bin Xu^[a]^, Menglu Chen^[a, b, c]^*, Kenan Zhang^[b]^, Jianbang Mu^[a]^, Jie Cao^[d,a]^*, Hongyu Lv^[a,b]^, Haoyu Zhang^[a,d]^, Xingting Zhou^[a,b]^, Feng Shi^[c]^, Qun Hao^[e,a]^*

[a] B. Xu, Prof. M. L. Chen, J. B. Mu, Prof. J. Cao, H. Y. Lv, H. Y. Zhang, X.T. Zhou, Prof. Q. Hao
School of Optics and Photonics, Beijing Institute of Technology
Beijing, 100081, China
Email: [menglu@bit.edu.cn](mailto:menglu@bit.edu.cn).

[b] Prof. M. L. Chen, K.N. Zhang, H.Y. Lv
Zhejiang Key Laboratory of 3D Micro/Nano Fabrication and Characterization, Westlake Institute for Optoelectronics

Zhejiang 311400, China.

[c] Prof. M. L. Chen, Prof. F. Shi

Laboratory of Science and Technology on Integrated Logistics Support

Changsha, Hunan 410073, China

[d] Prof. J. Cao, H.Y. Zhang

Yangtze Delta Region Academy of Beijing Institute of Technology,

Jiaxing 314019, China.

Email: caojie@bit.edu.cn

[e] Prof. Q. Hao
Physics Department, Changchun University of Science and Technology,
Changchun 130022, China.

**Supplementary Text**

**1. Materials**

The chemical compounds utilized in this study include cesium carbonate (Cs_2_CO_3_, 99.9%), lead chloride (PbCl_2_, 99.999%), lead(II) bromide (PbBr_2_, 99.999%), lead(II) iodide (PbI_2_, 99.999%), magnesium chloride (MgCl_2_, 98%), magnesium bromide (MgBr_2_, 98%), magnesium iodide (MgI_2_, 98%), 1-octadecene (ODE, 90%), oleic acid (OA, 90%), oleylamine (OAm, 90%), and toluene (AR). These chemical substances were sourced from Shanghai Aladdin Biochemical Technology Co., Ltd., ensuring a high level of purity for each reagent. In addition, SEBS, which refers to poly(styrene–ethylene–butylene–styrene), was acquired from Shenzhen Huixin Plastic Chemical Co. Ltd. It is important to note that all chemical reagents employed in this research were utilized in their received form without any additional purification processes, thereby maintaining their integrity and properties for the experiments conducted.

**2. Materials characterizations**

Transmission electron microscopy (TEM), high-resolution transmission electron microscopy (HRTEM), selected area electron diffraction (SAED), and energy-dispersive X-ray spectroscopy (EDS) of the Mg-CsPbX_3_ sample are detected by SU-8010. The X-ray powder diffraction (XRD) pattern was measured by X-ray diffraction using Cu-Kα radiation (Empyrean, Holland Panalytical). Optical absorption spectra were recorded by UV/Vis spectrophotometer (Lambda750, PerkinElmer). The photoluminescence (PL) and photoluminescence excitation (PLE) spectra are measured by a fluorescence indexer (F-7000, Hitachi). The time-resolved PL decay curves are detected by an FLS1000 spectrophotometer. Absolute PLQY is obtained by an FLS1000 fluorescence spectrophotometer. The X-ray photoelectron spectra (XPS) spectra were measured with X-ray photoelectron spectroscopy (Thermo Kalpha) with Al Kα radiation of 1486 eV and all the XPS data were checked according to the C 1s binding energy of 284.6 eV. The thickness of the Mg-CsPbX_3_@SEBS film is obtained by a stylus profiler (Alpha-Step D-300).

**3.** **Material synthesis**

**3.1 Preparation of a Cs-oleate precursor**

A total of 0.814 g of Cs_2_CO_3_ was placed into a 50 mL three-necked flask along with 40 mL of 1-octadecene and 2.5 mL of oleic acid. The mixture underwent magnetic stirring under a nitrogen (N_2_) environment, was dried for 1 hour at 120 °C to remove both moisture and oxygen, and subsequently heated to 150 °C in the N_2_ atmosphere until all Cs_2_CO_3_ fully reacted with oleic acid. Before proceeding with the preparation of CsPbBr_3_ PQDs, the solution was heated further at 100 °C since Cs-oleate begins to precipitate from 1-octadecene at room temperature.

**3.2 Synthesis of CsPbX_3_ PQDs**

The synthesis of all-inorganic CsPbX_3_ (where X = Cl, Br, I) perovskite quantum dots (PQDs) was conducted through a hot-injection technique. Under a nitrogen atmosphere in a 50 mL three-necked flask, a mixture comprising 5 mL of 1-octadecene, 0.5 mL of oleic acid, 0.5 mL of oleylamine, and 0.188 mmol of PbX_2_—specifically, 0.087 g of PbI_2_, 0.069 g of PbBr_2_, 0.052 g of PbCl_2_, or combinations thereof—was heated to 120 °C for one hour. Following this, the temperature was increased to 160 °C and held constant for 10 minutes prior to swiftly injecting a hot solution of the Cs-oleate precursor, prepared as previously described, into the flask within a 5-second window. The reaction mixture was subsequently cooled with an ice-water bath until condensation was noted. The resultant crude solution underwent centrifugation at 10,000 rpm for 5 minutes at 20 °C, after which the supernatant was discarded, and the precipitate of CsPbBr3 PQDs was dispersed in 10 mL of cyclohexane to yield a stable long-term solution.

**3.3 Preparation of MgX_2_ stock solution**

0.30 mmol MgX_2_ (0.055 g MgBr_2_ or 0.029 g MgCl_2_ ) was dissolved in 4 mL OAm by heating the mixture at 120 °C for 2h to obtain an opaque solution of MgX_2_-OAm.

**3.4** **Post-synthetic processing of the synthesized Mg-CsPbX_3_ PQDs**

An aliquot of 5 µL from the previously mentioned solution was diluted with 50 µL of hexane. Following this, 30 µL of the diluted solution was combined with a stirred suspension (550 µL) of CsPbX_3_ NCs in hexane, which was situated in a 25 ml single-neck round-bottom flask equipped with a magnetic stir bar. The mixture was stirred for one hour at room temperature, which has been identified as the ideal time frame for effectively completing the treatment. Afterward, a portion of 500 µL from the resulting solution was extracted and combined with 1 mL of methyl acetate, then it was promptly centrifuged at 8,000 rpm for 5 minutes. The supernatant, which contained excess ligands and salts, was discarded, leaving behind a small residue of purified NCs that settled at the base and was subsequently resuspended in hexane. These are high-quality Mg-doped CsPbX_3_ PQDs characterized by their bright emission and outstanding stability in ambient conditions.

**3.5 Fabrication of Mg-CsPbX_3_@SEBS composite films**

Composite films of Mg-CsPbX_3_ PQDs and SEBS were created by combining Mg-CsPbX_3_ PQDs with a solution of SEBS in cyclohexane, with concentrations varying between 0.03 g mL^−1^ and 0.15 g mL^−1^. The SEBS block copolymer was mixed into cyclohexane and allowed to dissolve completely at room temperature before introducing the dispersion of Mg-CsPbX_3_ PQDs. In this SEBS/cyclohexane mixture, the concentration of Mg-CsPbX_3_ PQDs was established at 0.25 mL mL^−1^. The components were thoroughly blended to achieve a homogenous dispersion. Next, a droplet of this blend was applied near the surface of the aqueous phase using a micropipette. The droplet quickly spread across the water's surface, resulting in the formation of a film as the cyclohexane evaporated.

**3.6 Computational details**

Quantum chemical studies are performed using density functional theory (DFT) implemented in GAUSSIAN 16 package^1^. Geometry optimization and frequency analysis are calculated at B3LYP hybrid functional^2^ with GD3BJ dispersion correlation at 6-311G(d, p) basis sets. Electrostatic potential surfaces (ESP) diagram is performed by Multiwfn 3.8^3^ and VMD v 1.9.3^4^ molecular visualization software.

The crystals of SEBS and PE were optimized using the Cambridge Sequential Total Energy Package (CASTEP)^5^ based on the pseudopotential plane wave (PPW) method. A plane-wave basis set was employed to expand the wave functions with a cutoff kinetic energy of 400 eV. For the electron-electron exchange and correlation interactions, the functional parametrized by Perdew-Burke-Ernzerhof (PBE), a form of the general gradient approximation (GGA), was used throughout. The convergence criterion for the electronic self-consistent field (SCF) loop was set to 2×10^-6^ eV/atom. The atomic structures were optimized until the residual forces were below 0.05 eVÅ^-1^. The bonding energy of Mg-CsPbBr_3_-SEBS is calculated as follows: E_b_ = E(AB) - E(A) - E(B).

1. Frisch, M., Trucks, G., Schlegel, H., Scuseria, G., Robb, M., Cheeseman, J., Scalmani, G., Barone, V., Petersson, G., and Nakatsuji, H. (2016). Gaussian 16, Revision C. 01 Normal name order. Gaussian.

2. Stephens, P.J., Devlin, F.J., Chabalowski, C.F., and Frisch, M.J. (1994). Ab initio calculation of vibrational absorption and circular dichroism spectra using density functional force fields. The Journal of physical chemistry *98*, 11623-11627.

3. Lu, T., and Chen, F. (2012). Multiwfn: a multifunctional wavefunction analyzer. Journal of computational chemistry *33*, 580-592.

4. Humphrey, W., Dalke, A., and Schulten, K. (1996). VMD: visual molecular dynamics. Journal of molecular graphics 14, 33-38.

5. Accelerys Materials Studio Release Notes, Release 5.5.1; Accelrys Software, Inc.: San Diego, 2010

**Supplementary Figures**


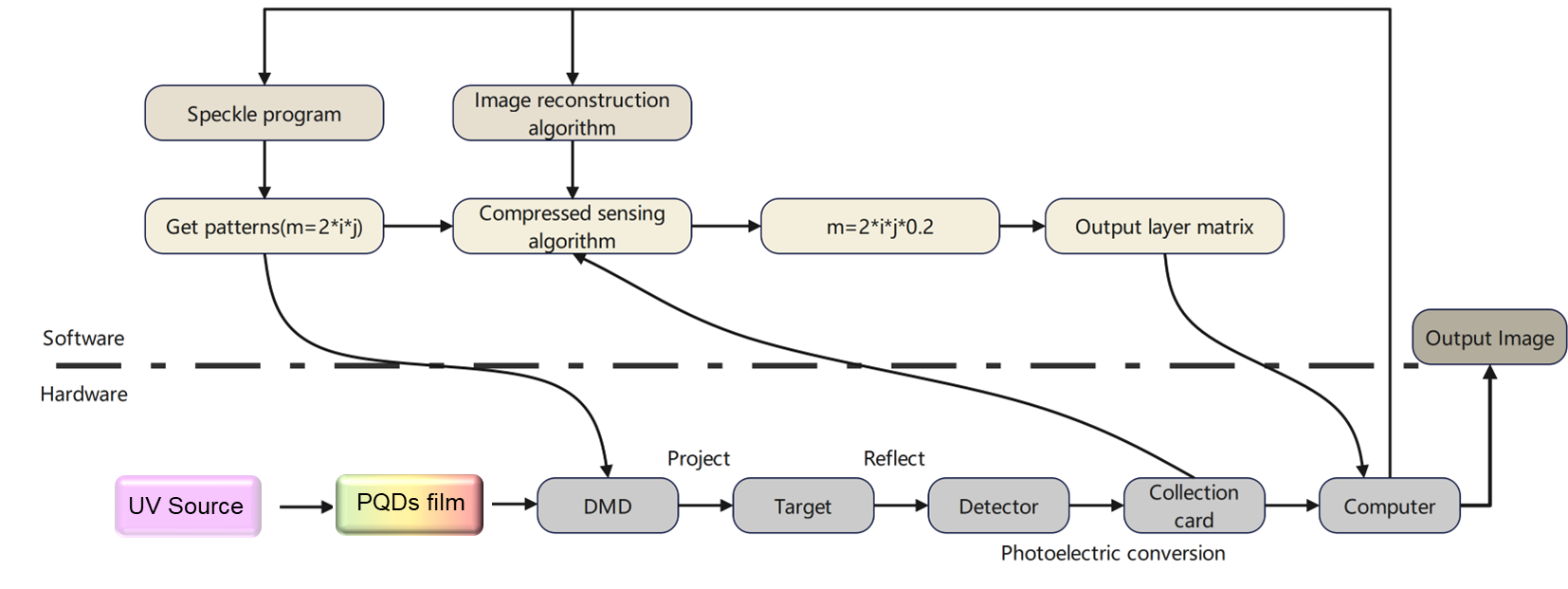


**Figure S1.** Basic software and hardware integration in the image reconstruction process of Fourier single pixel imaging.

In the section concerning light modulation, the visible light emitted from the Perovskite films undergoes modulation via a DMD (ViALUX V-7001). Illumination of the objects occurs through light that traverses a lens with a focal length of 125 mm. The distance between the lens and the projection module (Aunion Tech) for the DMD is established at 25 mm and 140 mm. For the receiving mechanism, a bucket detector (Thorlabs PDA100A) captures the light reflected off the objects at a distance of 90 mm. A data acquisition device (PicoScope-6404E) gathers the signals from the bucket detector. An Intel Xeno(R) Gold 6226R CPU paired with an Nvidia RTX 2080Ti GPU serves as the platform for executing the algorithms. The Compressed sensing algorithm in our Single pixel imaging framework is developed using Matlab. The Compressed sensing algorithm in our Single pixel imaging framework are developed using in Matlab. Within Fourier Single pixel imaging, We achieve using acquisition rates of 20% or even lower to recongsruct target image. For images of I × j, compress the required number of patterns from 2 × I × J to 2 × i × j × 0.2. Perform autocorrelation operation between the generated patterns and the light intensity collected by the detector to obtain colorful image of the target.


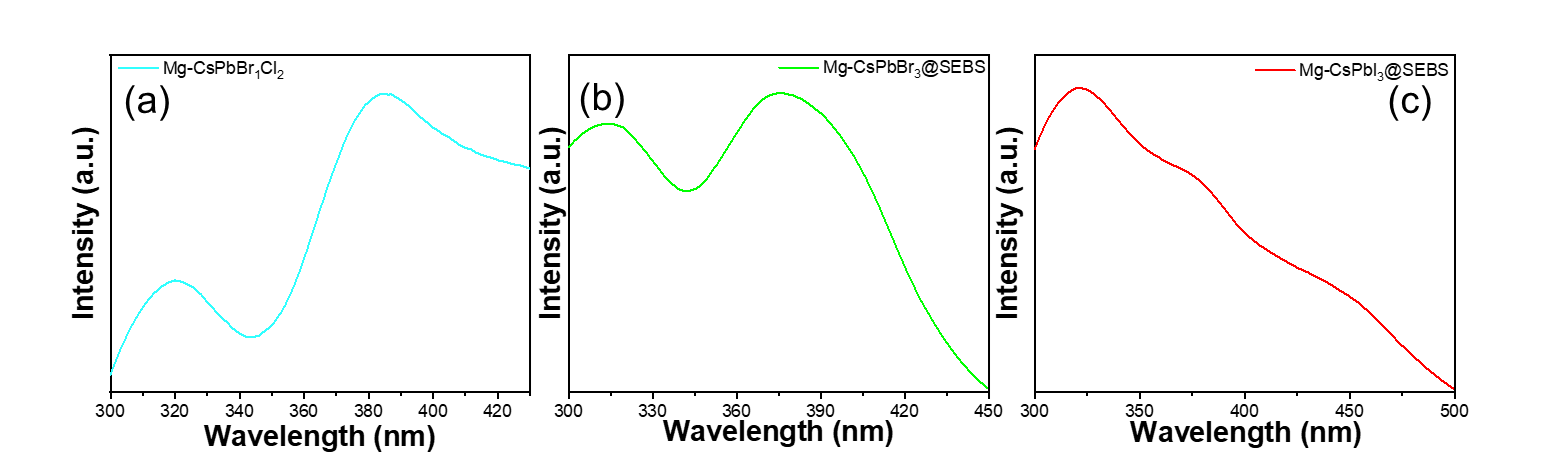


**Figure S2.** Fluorescence excitation spectra of Mg-CsPbX_3_@SEBS (a), Mg-CsPbBr_1_Cl_2_@SEBS (b), Mg-CsPbBr_3_@SEBS (c) Mg-CsPbI_3_@SEBS.


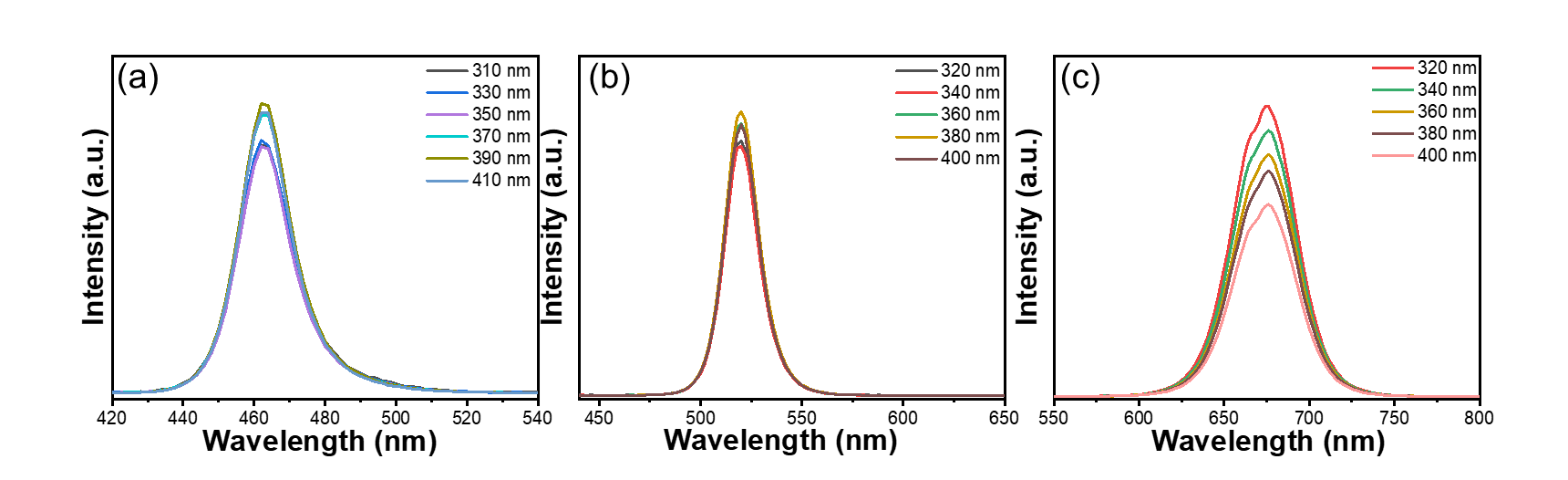


**Figure S3.** Evolution of the fluorescence spectra with the excitation wavelength for the Mg-CsPbX_3_@SEBS (a), Mg-CsPbBr_1_Cl_2_@SEBS (b), Mg-CsPbBr_3_@SEBS (c) Mg-CsPbI_3_@SEBS.


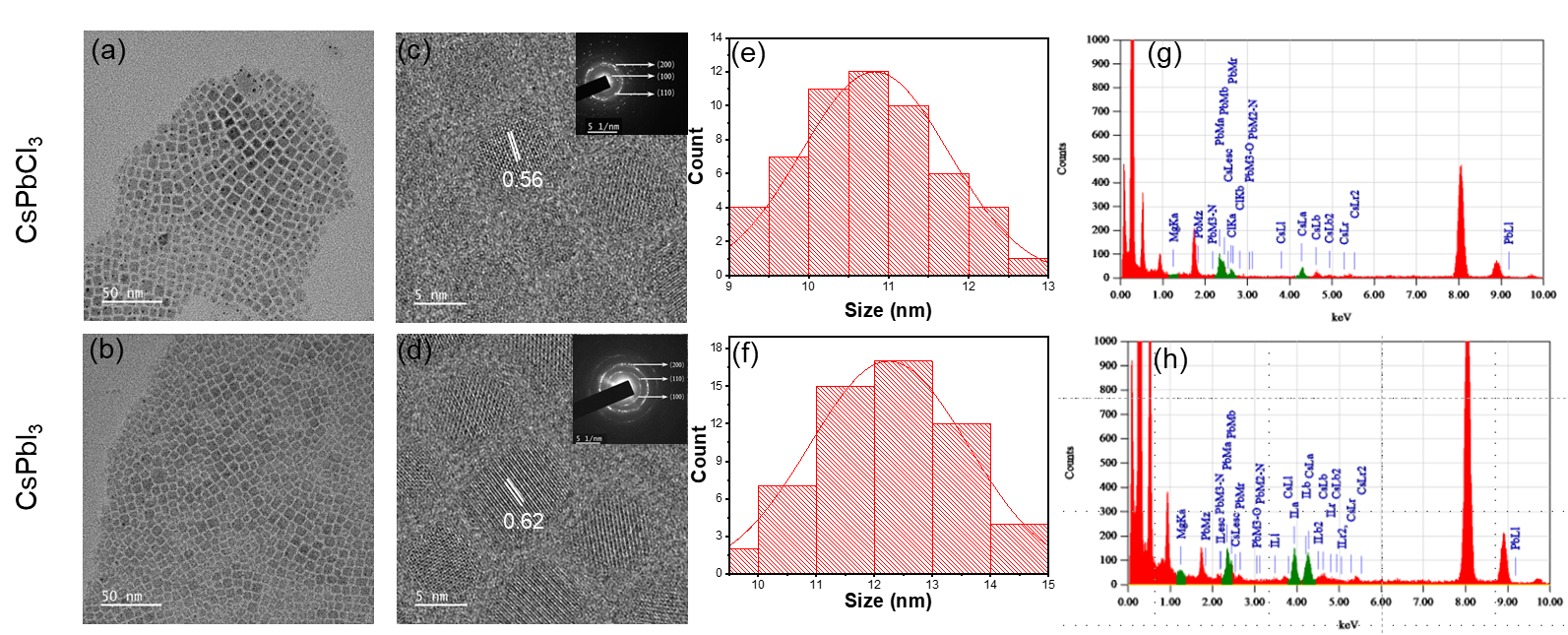


**Figure S4.** (a-b) TEM images; (c-d) High-resolution TEM (HRTEM) image and selected area electron diffraction (SAED) patterns; e-f) histograms showing size distribution; and (g-h) energy dispersive X-ray spectra for (a,c,e,g) CsPbCl_3_, (b,d,f,h) CsPbI_3_, The HRTEM images illustrate distinct lattice fringes, with measured d-spacings of 0.56 nm, and 0.62 nm corresponding to CsPbCl_3_, and CsPbI_3_ PQDs, respectively. These measurements align well with the (100) plane lattice spacings of cubic phase CsPbX_3_. The pronounced SAED ring patterns corroborate the high crystallinity and pure phase of the PQDs. Analysis of the energy dispersive X-ray spectra verifies the presence of elements Cs, Pb, and X (where X = Cl, and I) within CsPbX_3_ PeQDs. The size distribution histograms for the PQDs were derived from randomly assessing 200 particles within their TEM images.


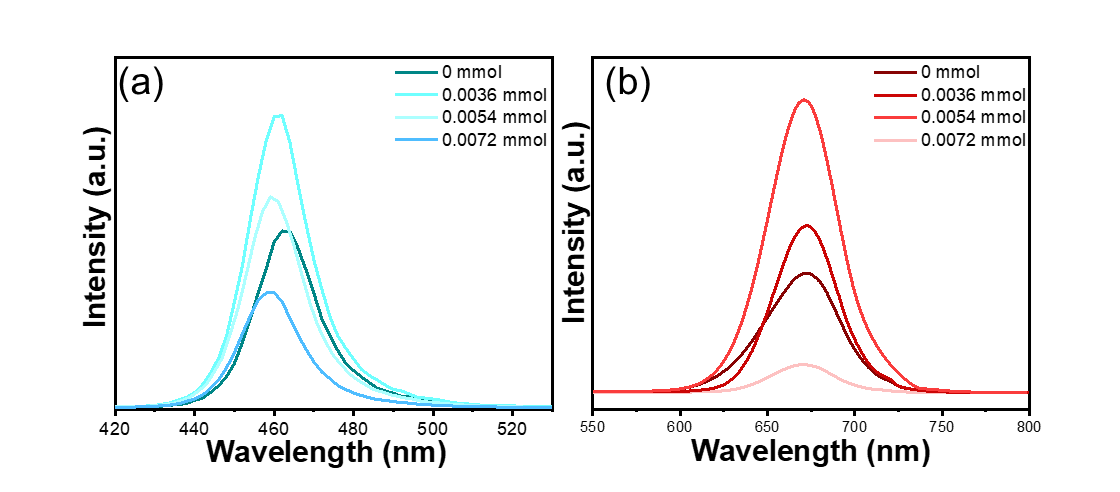


**Figure S5.** PL emission spectra of CsPbX_3_ doped with Mg^2+^ at different concentrations, (a) CsPbBr_1_Cl_2_ (b) CsPbI_3_.


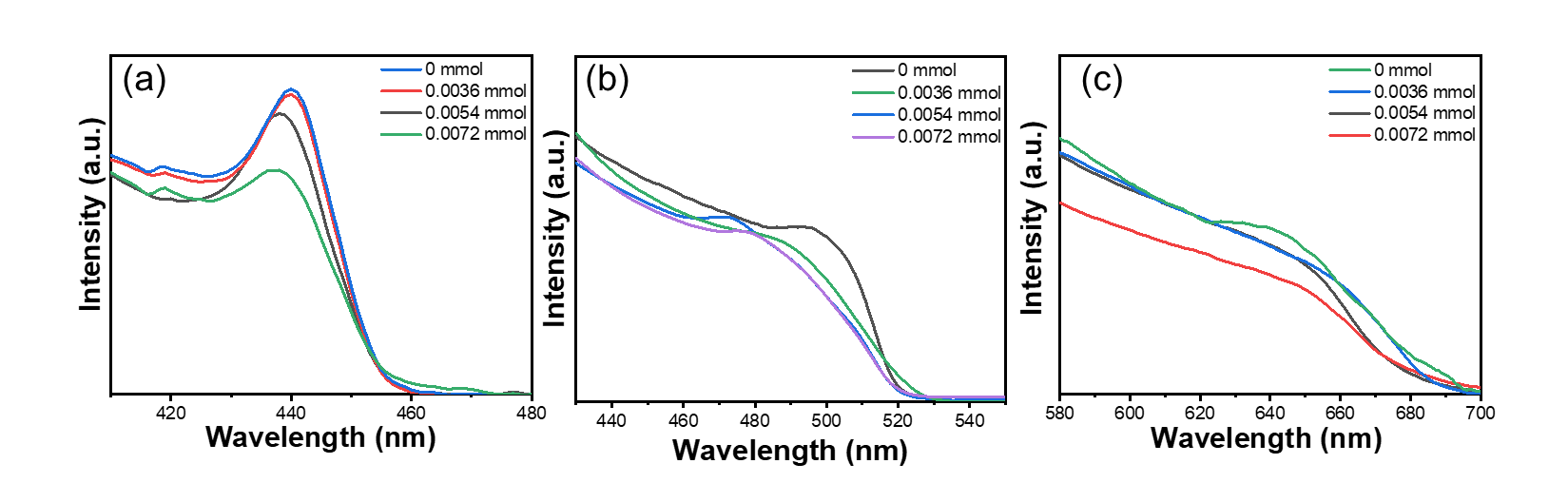


**Figure S6.** UV-vis optical absorption spectra of CsPbX_3_ doped with Mg^2+^ at different concentrations, (a) CsPbBr_1_Cl_2_ (b) CsPbBr_3_ (c) CsPbI_3_.

**Figure S7.** Time-resolved PL spectra of Mg-CsPbX_3_@SEBS (a), Mg-CsPbBr_1_Cl_2_@SEBS (b), Mg-CsPbI_3_@SEBS.


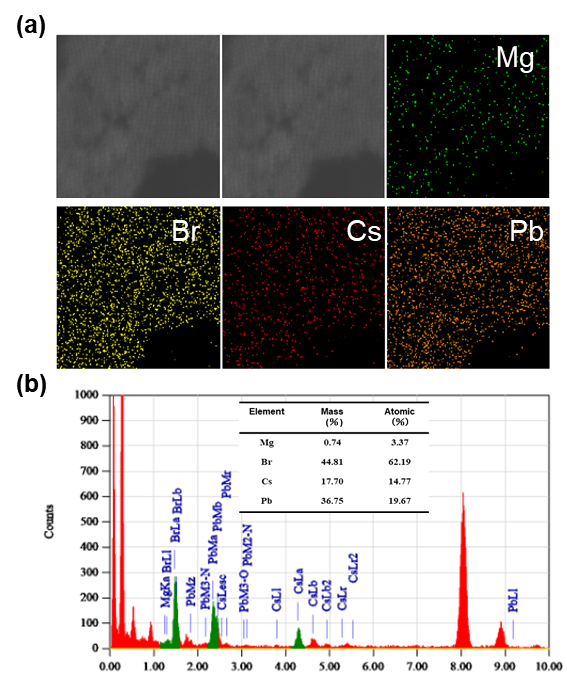


**Figure S8.** (a) Energy-dispersive spectrometry (EDS) elemental mappings of Mg-CsPbBr_3_ composite. (b) EDS spectra were taken from highlighted region of Mg-CsPbBr_3_ composite. The inset table shows the Mg, Cs, Pb and Br atomic ratio analysis results.


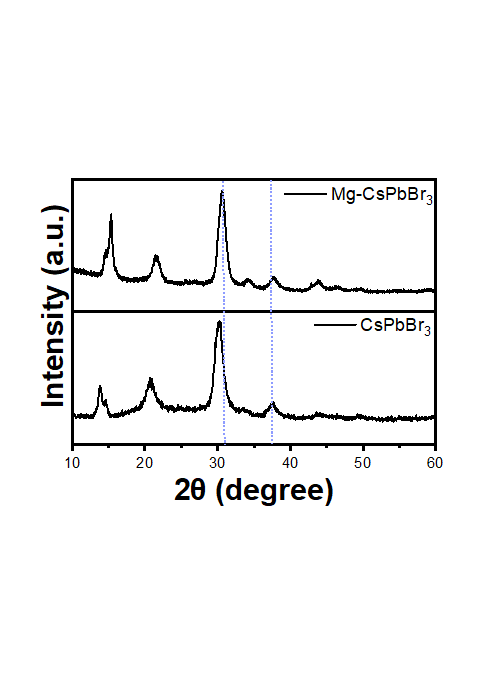


**Figure S9.** The XRD patterns of CsPbBr_3_ and Mg-CsPbBr_3_.

**Figure S10.** The size distributions of Mg-CsPbX_3_, (a) Mg-CsPbBr_1_Cl_2_ (b) Mg-CsPbI_3_


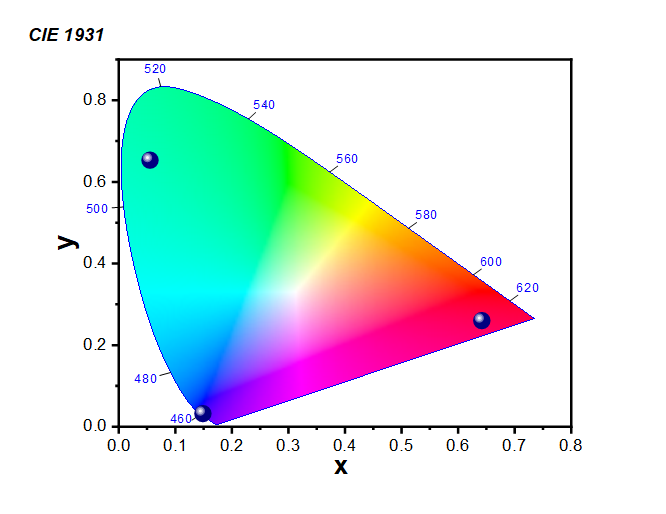


**Figure S11.** CIE coordinate diagram of PL spectra of Mg-CsPbBr_1_Cl_2_@SEBS, Mg-CsPbBr_3_@SEBS and Mg-CsPbI_3_@SEBS.


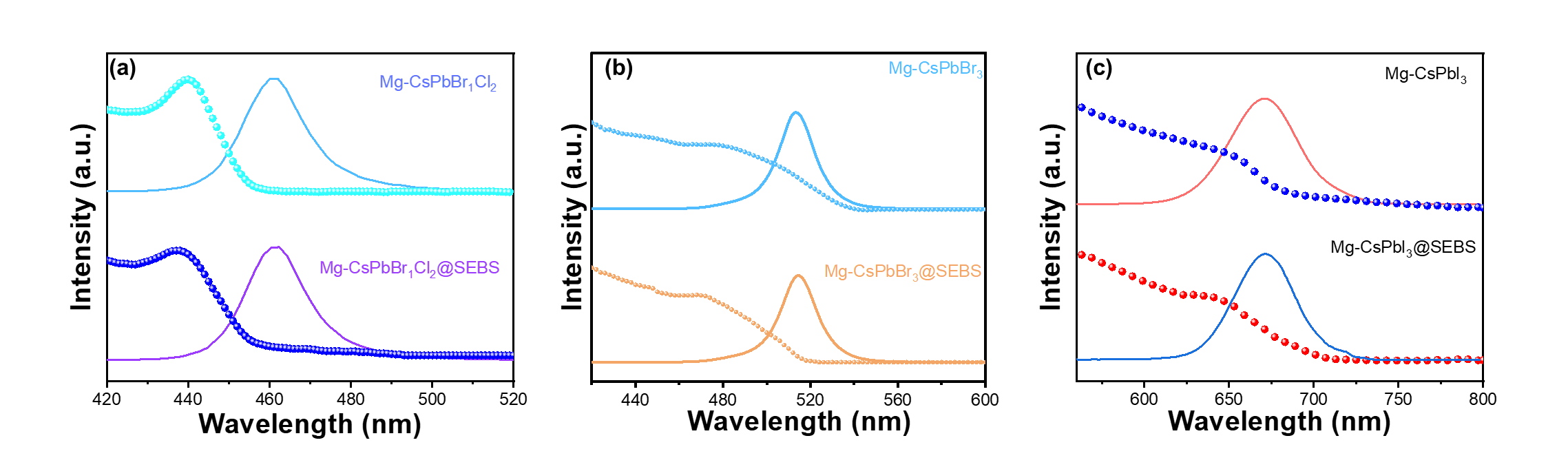


**Figure S12.** PL spectra and UV-visible absorption of Mg-CsPbX_3_ PQDs and Mg-CsPbX_3_@SEBS Film (a), Mg-CsPbBr_1_Cl_2_ and Mg-CsPbBr_1_Cl_2_@SEBS (b), Mg-CsPbBr_3_ and Mg-CsPbBr_3_@SEBS (c), Mg-CsPbI_3_ and Mg-CsPbI_3_@SEBS.

.


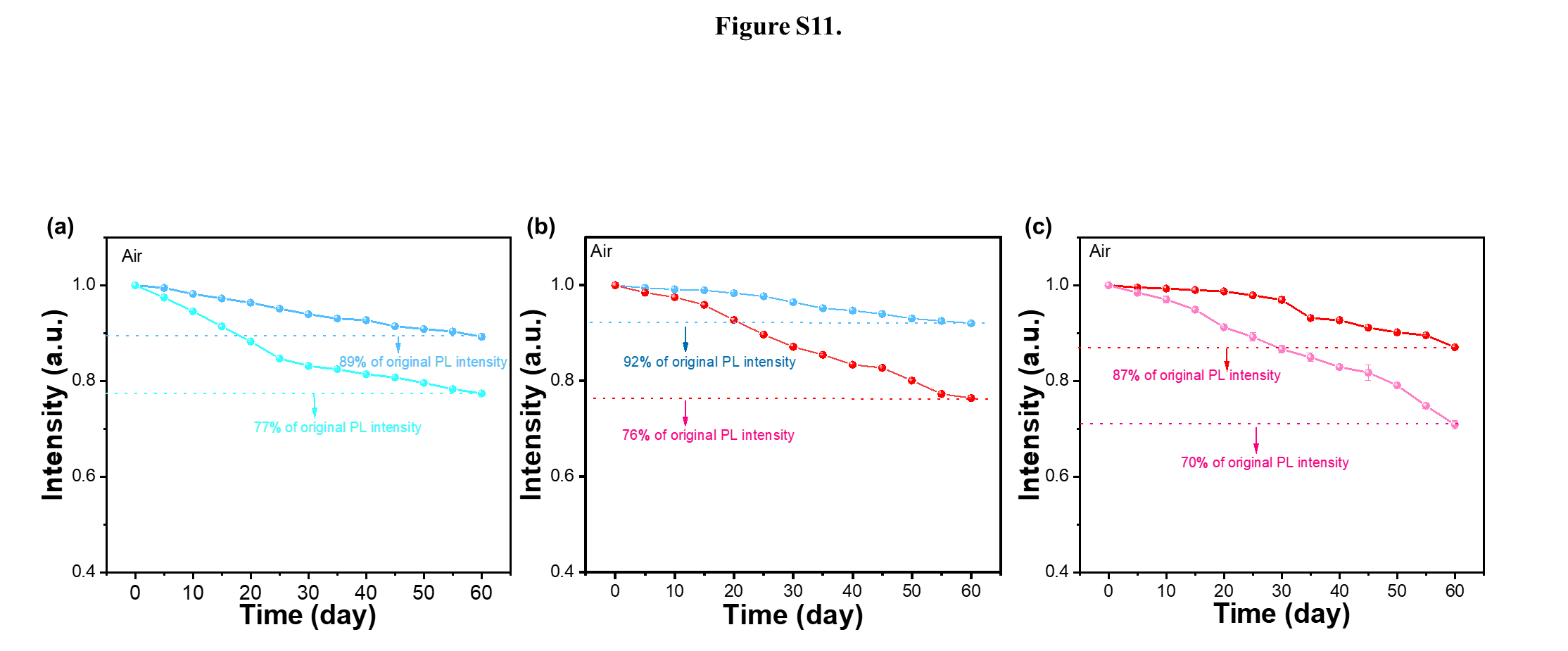


**Figure S13.** PL intensity of Mg-CsPbX_3_ PQDs and Mg-CsPbX_3_@SEBS Film stored at ambient conditions (temperature: 25 °C, humidity: 60–70%). (a), Mg-CsPbBr_1_Cl_2_ and Mg-CsPbBr_1_Cl_2_@SEBS (b), Mg-CsPbBr_3_ and Mg-CsPbBr_3_@SEBS (c), Mg-CsPbI_3_ and Mg-CsPbI_3_@SEBS.


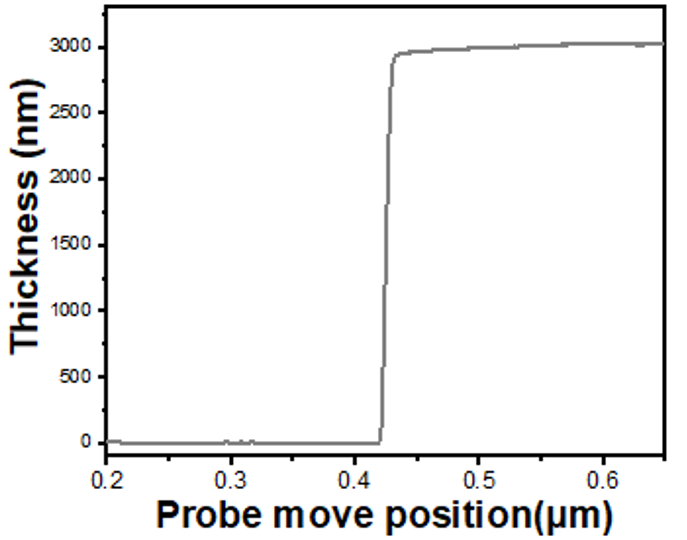


**Figure S14.** The step profiler measurement of Mg-CsPbBr_3_@SEBS film.


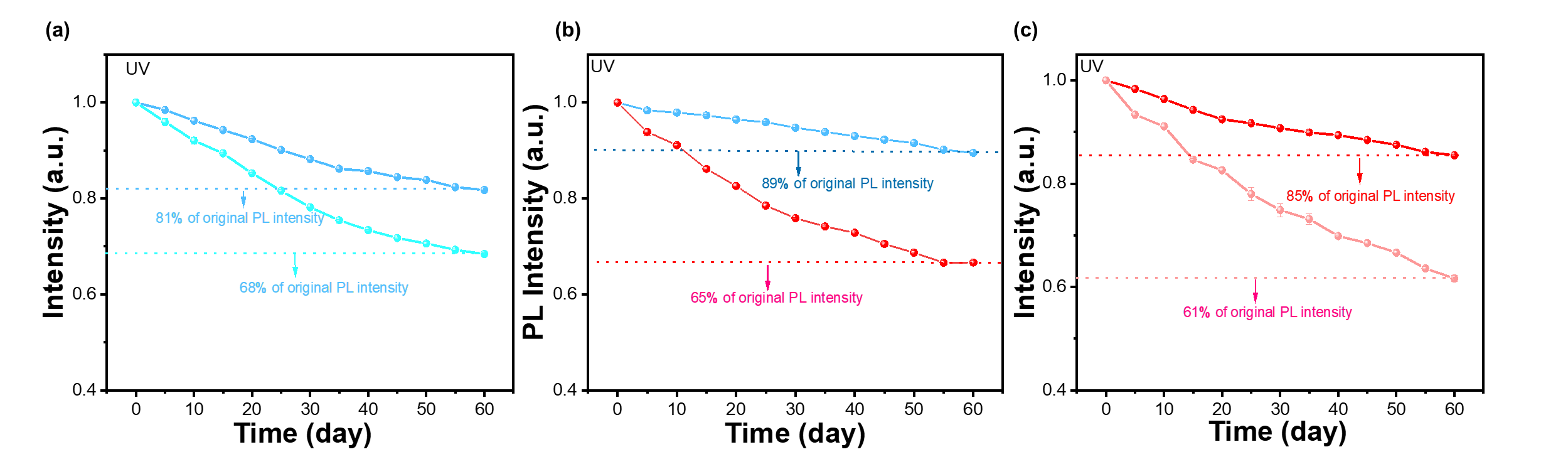


**Figure S15.** PL intensity of Mg-CsPbX_3_ PQDs and Mg-CsPbX_3_@SEBS Film under UV light irradiation (𝜆 = 365 nm, 15 mW cm^−2^). (a), Mg-CsPbBr_1_Cl_2_ and Mg-CsPbBr_1_Cl_2_@SEBS (b), Mg-CsPbBr_3_ and Mg-CsPbBr_3_@SEBS (c), Mg-CsPbI_3_ and Mg-CsPbI_3_@SEBS.


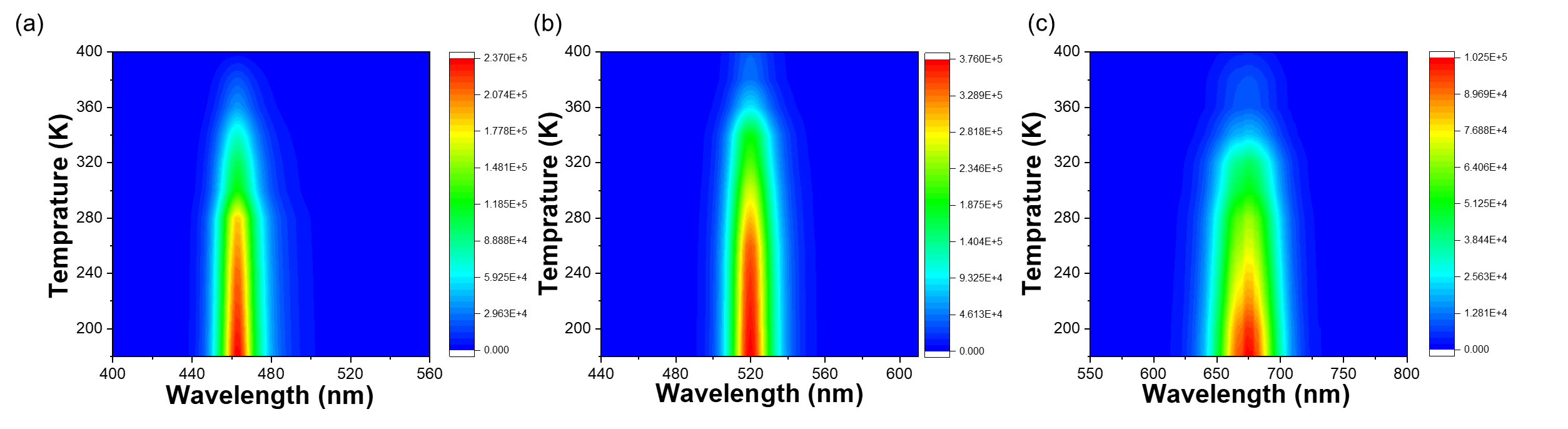


**Figure S16.** PL spectra of CsPbX_3_ at different temperatures. (a), Mg-CsPbBr_1_Cl_2_@SEBS (b), Mg-CsPbBr_3_@SEBS (c), Mg-CsPbI_3_@SEBS.


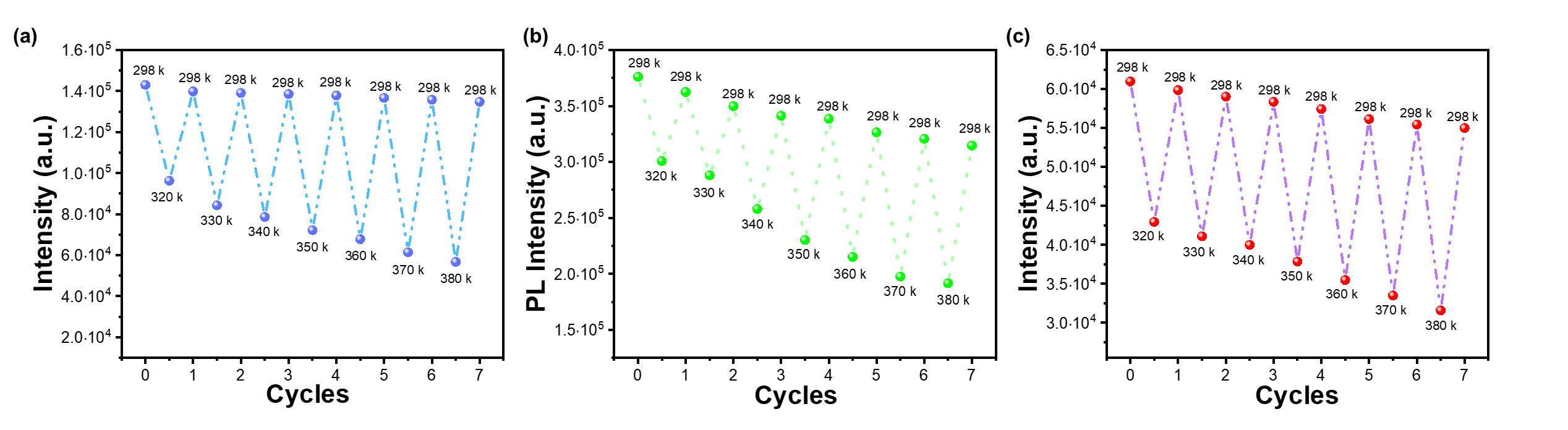


**Figure S17.** PL spectral intensity of (a), Mg-CsPbBr_1_Cl_2_@SEBS (b), Mg-CsPbBr_3_@SEBS (c), Mg-CsPbI_3_@SEBS films under heating/cooling cycles.


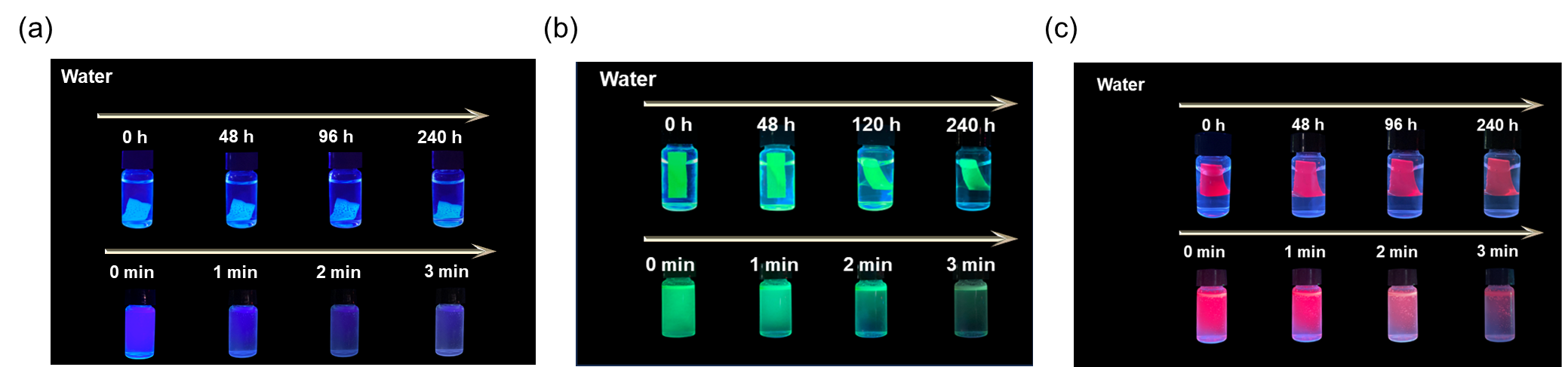


**Figure S18.** Optical photographs of Mg-CsPbX_3_ PQDs and Mg- CsPbX_3_@SEBS films dispersed in aqueous solution. (a) Mg-CsPbBr_1_Cl_2_ and Mg-CsPbBr_1_Cl_2_@SEBS (b), Mg-CsPbBr_3_ and Mg-CsPbBr_3_@SEBS (c), Mg-CsPbI_3_ and Mg-CsPbI_3_@SEBS.


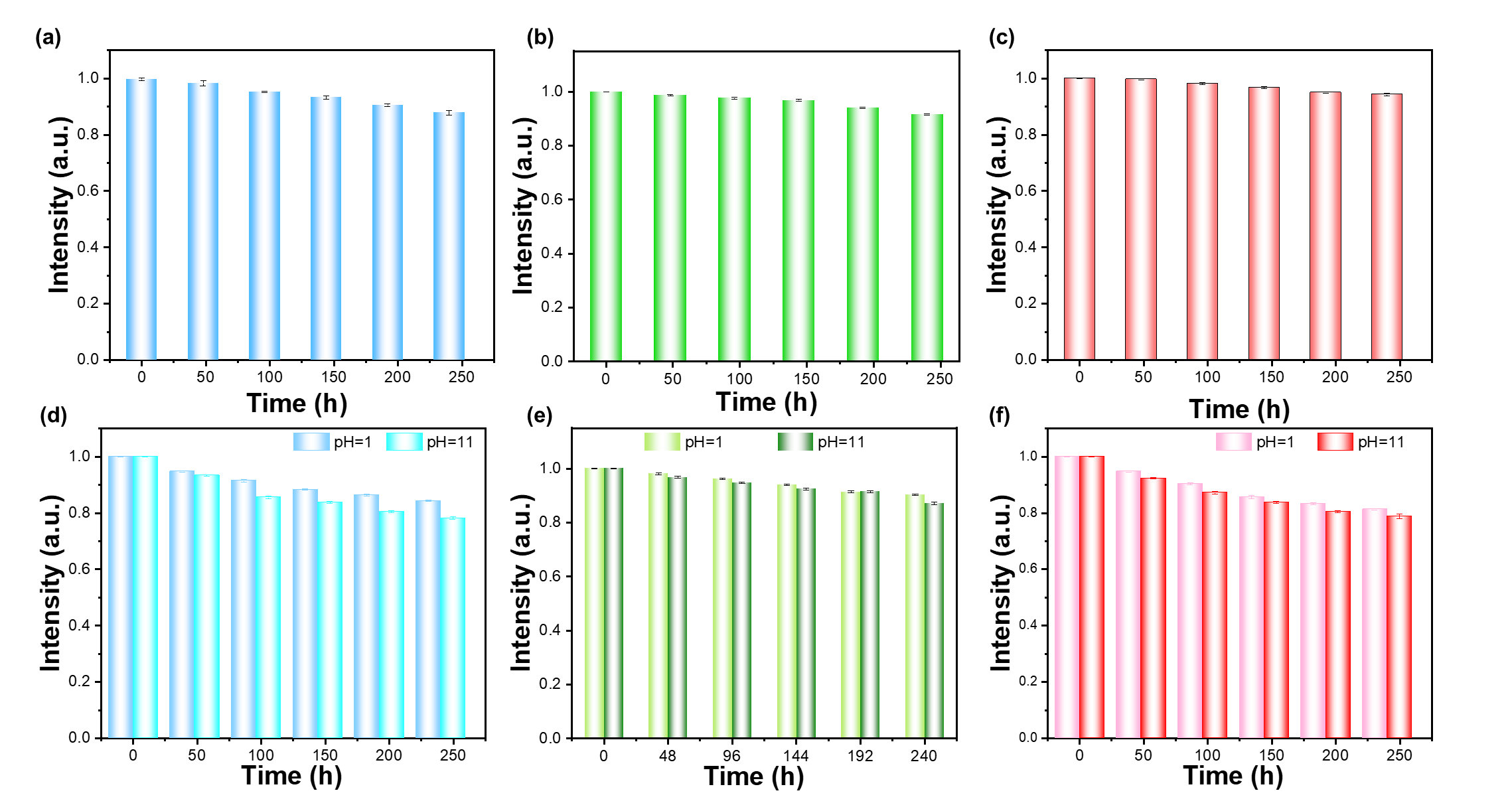


**Figure S19.** Relative PL intensities corresponding to Mg-CsPbBr_1_Cl_2_@SEBS, Mg-CsPbBr_3_@SEBS and Mg-CsPbI_3_@SEBS films dispersed in a-c) water, and d-f) aqueous solutions at pH 1 and pH 11, respectively.


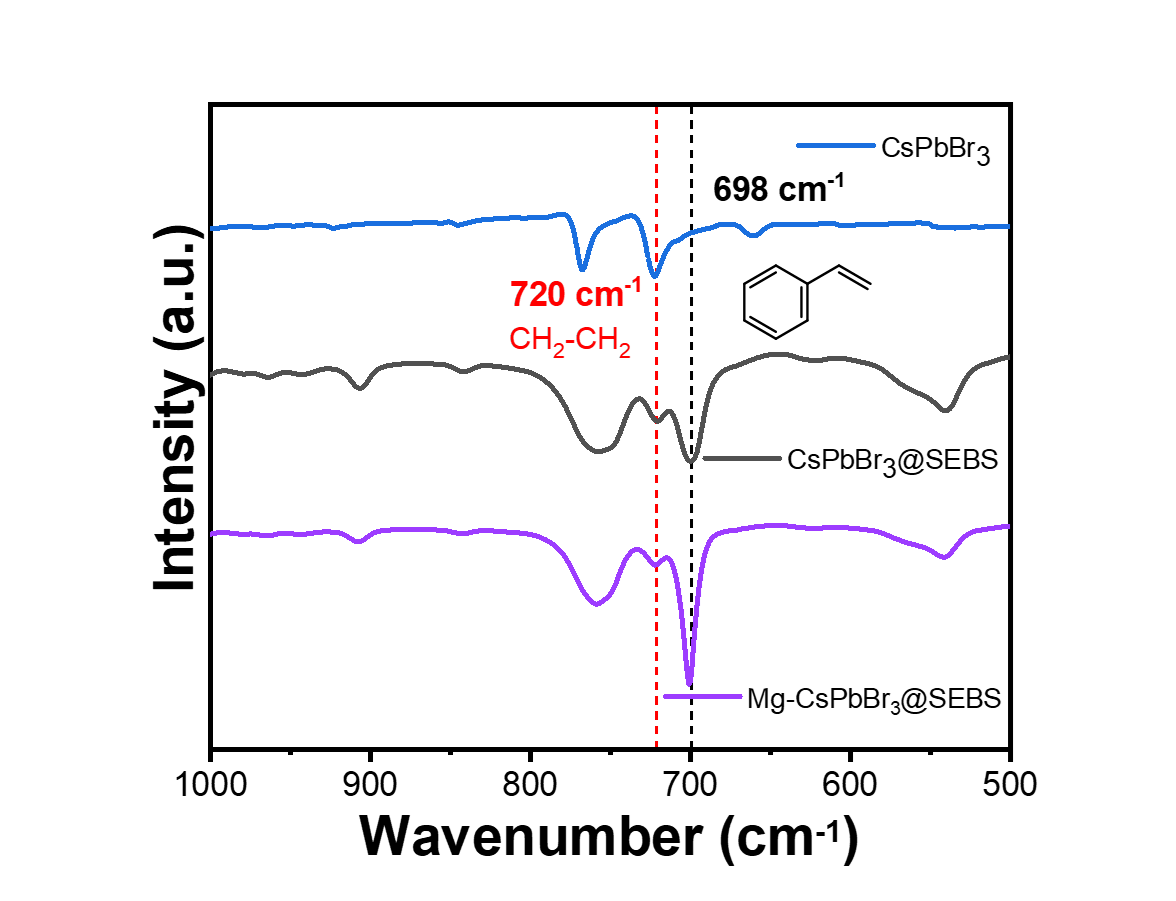


**Figure S20.** FTIR spectra of CsPbBr_3_, CsPbBr_3_@SEBS, and Mg-CsPbBr_3_@SEBS.


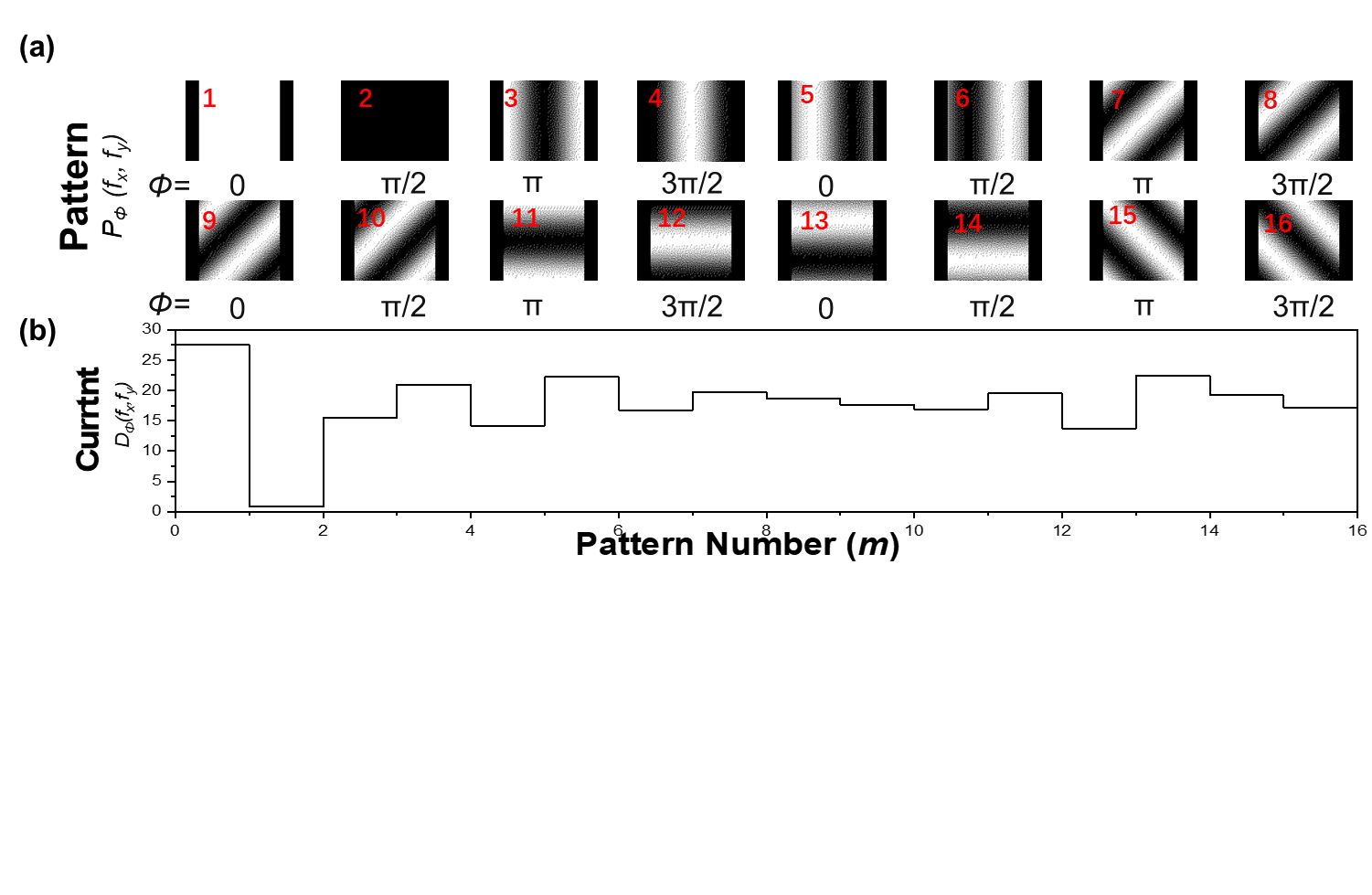


**Figure S21.** (a) The the first fouth groups patterns generated by four steps phase shifted (b) The intensity of the two-dimensional light field collected by detector when corresponding pattern is projected to the target.


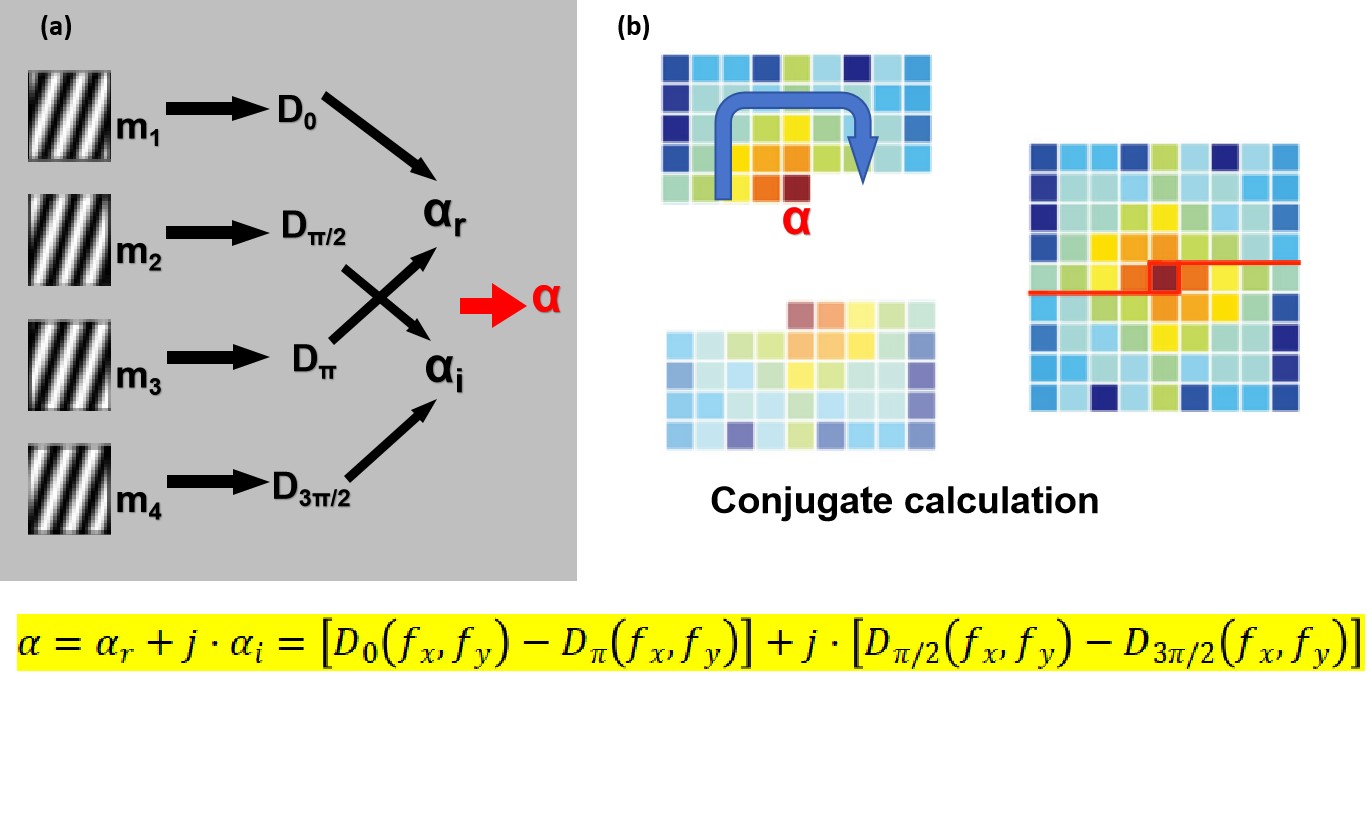


**Figure S22.** The detector collects a set of fourth-order phase-shifted speckle projections and obtains D*_Φ_*. The spectral intensity α is calculated by the formula:$a=a_{r}+j\cdot a_{i}=\left[ D_{0}\left( f_{x},f_{y} \right)-D_{\pi}\left( f_{x},f_{y} \right) \right]+j\cdot\left[ D_{\pi/2}\left( f_{x},f_{y} \right)-D_{3\pi/2}\left( f_{x},f_{y} \right) \right]$ differential.

**Supplementary Tables**

**Table S1** Fitting parameters of the phosphorescence decay curves of CsPbCl_3_, CsPbBr_1_Cl_2_, CsPbBr_2_Cl_1_, CsPbBr_3_, CsPbBr_2_I_1_, CsPbBr_1_I_2_, and CsPbI_3_.

| **Sample** | **τ_1_ (ns)** | **A_1_** | **τ_2_ (ns)** | **A_2_** | **τ_avg_ (ns)** | **x^2^** |
| --- | --- | --- | --- | --- | --- | --- |
| **CsPbCl_3_** | 4.89 | 0.22 | 8.72 | 0.78 | 7.31 | 0.999 |
| **CsPbBr_1_Cl_2_** | 3.36 | 0.20 | 15.71 | 0.80 | 13.24 | 0.999 |
| **CsPbBr_2_Cl_1_** | 4.41 | 0.18 | 20.52 | 0.82 | 17.62 | 0.999 |
| **CsPbBr_3_** | 7.55 | 0.17 | 26.72 | 0.83 | 23.46 | 0.999 |
| **CsPbBr_2_I_1_** | 12.39 | 0.16 | 37.21 | 0.84 | 33.24 | 0.999 |
| **CsPbBr_1_I_2_** | 87.94 | 0.18 | 60.28 | 0.82 | 65.26 | 0.999 |
| **CsPbI_3_** | 47.81 | 0.12 | 121.14 | 0.88 | 112.34 | 0.999 |

**Table S2** Fitting parameters of the phosphorescence decay curves of CsPbCl_3_, CsPbBr_1_Cl_2_, CsPbBr_2_Cl_1_, CsPbBr_3_, CsPbBr_2_I_1_, CsPbBr_1_I_2_, and CsPbI_3_.

| **Sample** | | **PL Wavelength**  **(nm)** | **PLQY** | | |
| --- | --- | --- | --- | --- | --- |
|  |  |  | **CsPbX_3_**  **solution** | **Mg-CsPbX_3_ solution** | **Mg-CsPbX_3_@SEBS**  **film** |
| **CsPbBr_3_** | 519 | | 64.23 | 95.99 | 83.6 |
| **CsPbI_3_** | 676 | | 69.4 | 96.01 | 88.11 |
| **CsPbBr_1_Cl_2_** | 462 | | 50.29 | 87.64 | 81.73 |

**Table S3** Fitting parameters of the phosphorescence decay curves of Mg-CsPbCl_3_, Mg-CsPbBr_3_, and Mg-CsPbI_3_.

| **Sample** | **τ_1_ (ns)** | **A_1_** | **τ_2_ (ns)** | **A_2_** | **τ_avg_(ns)** | **x^2^** |
| --- | --- | --- | --- | --- | --- | --- |
| **Mg-CsPbBr_1_Cl_2_** | 11.10 | 0.28 | 17.82 | 0.72 | 15.94 | 0.999 |
| **Mg-CsPbBr_3_** | 23.54 | 0.21 | 46.21 | 0.79 | 41.51 | 0.999 |
| **Mg-CsPbI_3_** | 75.76 | 0.19 | 145.39 | 0.81 | 132.16 | 0.999 |
